# Decreased NHE3 expression in colon cancer is associated with DNA damage, increased inflammation and tumor growth

**DOI:** 10.1038/s41598-022-19091-x

**Published:** 2022-08-30

**Authors:** Daniel Laubitz, Michael A. Gurney, Monica Midura-Kiela, Christy Clutter, David G. Besselsen, Hao Chen, Fayez K. Ghishan, Pawel R. Kiela

**Affiliations:** 1 Department of Pediatrics, Steele Children’s Research Center, University of Arizona College of Medicine, 1501 N. Campbell Ave, Tucson, AZ 85724 USA; 2 University Animal Care, University of Arizona, Tucson, AZ USA; 3 Department of Pathology, University of Texas Southwestern Medical Center, Dallas, TX USA; 4 Department of Immunobiology, University of Arizona College of Medicine, Tucson, AZ USA

**Keywords:** Colorectal cancer, Cancer microenvironment, Gastrointestinal diseases

## Abstract

Dysregulation of intra- and extracellular pH in cancer contributes to extracellular matrix remodeling, favors cell migration, proliferation, and metastasis. Although the primary attention has been focused on the role of the ubiquitous Na^+^/H^+^ exchanger isoform NHE1, the role of NHE3, the predominant apical isoform in colonic surface epithelium in the pathogenesis of colon cancer has not been investigated. Here, we show that NHE3 mRNA expression is significantly reduced in colorectal cancer patients and that low NHE3 expression is associated with poorer survival. Deletion of NHE3 in Apc^Min^ mice evaluated at 15 weeks of age (significant mortality was observed beyond this time) led to lower body weights, increased mucosal inflammation, increased colonic tumor numbers, evidence of enhanced DNA damage in tumor surface epithelium, and to significant alteration in the gut microbiota. In the absence of the inflammatory and microbial pressors, ca. 70% knockdown of NHE3 expression in SK-CO15 cells led to reduced intracellular pH, elevated apical pH, dramatic differences in their transcriptomic profile, increased susceptibility to DNA damage, increased proliferation, decreased apoptosis and reduced adhesion to extracellular matrix proteins. Our findings suggest that loss of NHE3 in the surface epithelium of colonic tumors has profound consequences for cancer progression and behavior.

## Introduction

Most cancer cells display resilience to metabolic stress resulting in part from increased glucose and nutrient uptake and enhanced lactic acid production. They effectively adapt to hypoxic and low-nutrient microenvironments created by abnormal vasculature, poor blood delivery, and impaired fluid clearance. Interstitial acidification and the concomitant intracellular alkalization are adaptive hallmarks of cancer, leading to a reversed pH gradient in cancer cells^1^. Prolonged extracellular acidification (low pH_e_) is thought to contribute to extracellular matrix remodeling and cancer metastasis, while intracellular alkalinization (elevated pH_i_) has been reported to favor cell proliferation^1^. Regulation of pH_e_ and pH_i_ in cancer is complex and involves dysregulation of expression and/or activity of several plasma membrane transporters that facilitate H^+^ efflux and of the cytosolic carbonic anhydrases. Considerable attention has been directed toward Na^+^/H^+^ exchanger 1 (NHE1; encoded by*SLC9A1*) which contributes to the increased intracellular H^+^ efflux from cancer cells^2,3^. NHE1-targeted therapy has been proposed as ancillary therapy to overcome the cancer cell metabolic resilience, and indeed, several in vitro studies demonstrated that NHE1 inhibition amplifies the efficiency of chemotherapeutics^4–6^, and mouse xenografts of NHE1-deficient cancer cell lines showed reduced capacity for growth^7,8^.

Colorectal cancer (CRC), the third most common cancer and the third leading cause of cancer-related deaths in the United States^9^, presents with additional layers of complications, such as cellular heterogeneity, epithelial barrier and mucosal immune system functions under the inflammatory pressure of the colonic microbiota, the associated pathobionts, and their metabolic products^10,11^. In the context of pH regulation, expression of multiple isoforms of Na^+^/H^+^ exchanger^12^ further complicates the dissection of factors contributing to initiation and progression of CRC. NHE3, encoded by *SLC9A3* gene, is the predominant apical Na^+^/H^+^ exchanger in the small intestinal and colonic epithelium. We and others have shown that NHE3 plays crucial roles in the intestinal sodium and water absorption, regulation of intracellular pH, gene expression, and contributes to microbial homeostasis in the gut^13–16^. NHE3^−/−^ mice develop spontaneous colitis that can be ameliorated with broad spectrum antibiotics^15^. It is consistent with the recently described role of inactivating *SLC9A3* mutations in the pathogenesis of very early onset IBD^17^. NHE3^−/−^ mice are highly susceptible to DSS-mediated mucosal injury and NHE3 deficiency exacerbates spontaneous colitis in IL-10^−/−^ mice^18,19^. Loss of NHE3 activity leads to microbial dysbiosis reminiscent of human IBD^20^, and in a model of adoptive T cell transfer, NHE3 status was the most significant determinant of gut microbial community^14^. In recent studies with gnotobiotic *RAG1*^−/−^ or *IL10*^−/−^ mice with fecal microbiota transfer from WT or NHE3-deficient mice, we demonstrated a causative relationship between NHE3-induced dysbiosis and colitis, with accelerated onset, and more severe inflammatory responses in mice colonized with microbiota from NHE3^−/−^ mice^21^.

These findings and the recognized role of inflammation and microbiota in the initiation and progression of CRC prompted us to investigate the expression and role of NHE3 in human colon cancer and in a defined mouse model of CRC. Among the genomic changes associated with CRC, loss-of-function mutations in the Apc (adenomatous polyposis coli) gene are the prevalent and are thought to represent the initiating event in ~ 80% of CRC occurrences^22^. The multiple intestinal neoplasia (Min) mouse (Apc^Min/+^) is a common animal model of intestinal carcinogenesis^23^. Although the spontaneous intestinal adenomas (small bowel and colonic) form with age spontaneously, inflammation enhances development of CRC in this model as demonstrated with dextran sulfate sodium (DSS)-induced colitis^24^, and in Apc^Min/+^ mice with deficient IL-10 signaling^25–27^. These findings, along with the reports highlighting the detrimental role of enteric bacteria in Apc^Min/+^ mice^26,28,29^ further support the notion that microbiota-driven inflammation underlies colitis-associated CRC.

In this report, we demonstrate that NHE3 expression is significantly reduced in sporadic CRC in human patients. We also show that loss of NHE3 accelerates the development of CRC and increases tumor burden in NHE3-deficient Apc^Min/+^ mice along with alteration of gut microbiota. We also demonstrate the intrinsic, microbial-independent consequences of NHE3 downregulation in human CRC cells such as increased susceptibility to DNA damage, increased proliferation, decreased apoptosis and adhesion to extracellular matrix proteins. These findings suggest that loss of NHE3 in the surface epithelium of colonic tumors may have profound consequences to cancer progression and behavior.

## Results

### Human NHE3 mRNA is downregulated in colorectal cancer and correlates with patient survival

**Figure 1:**
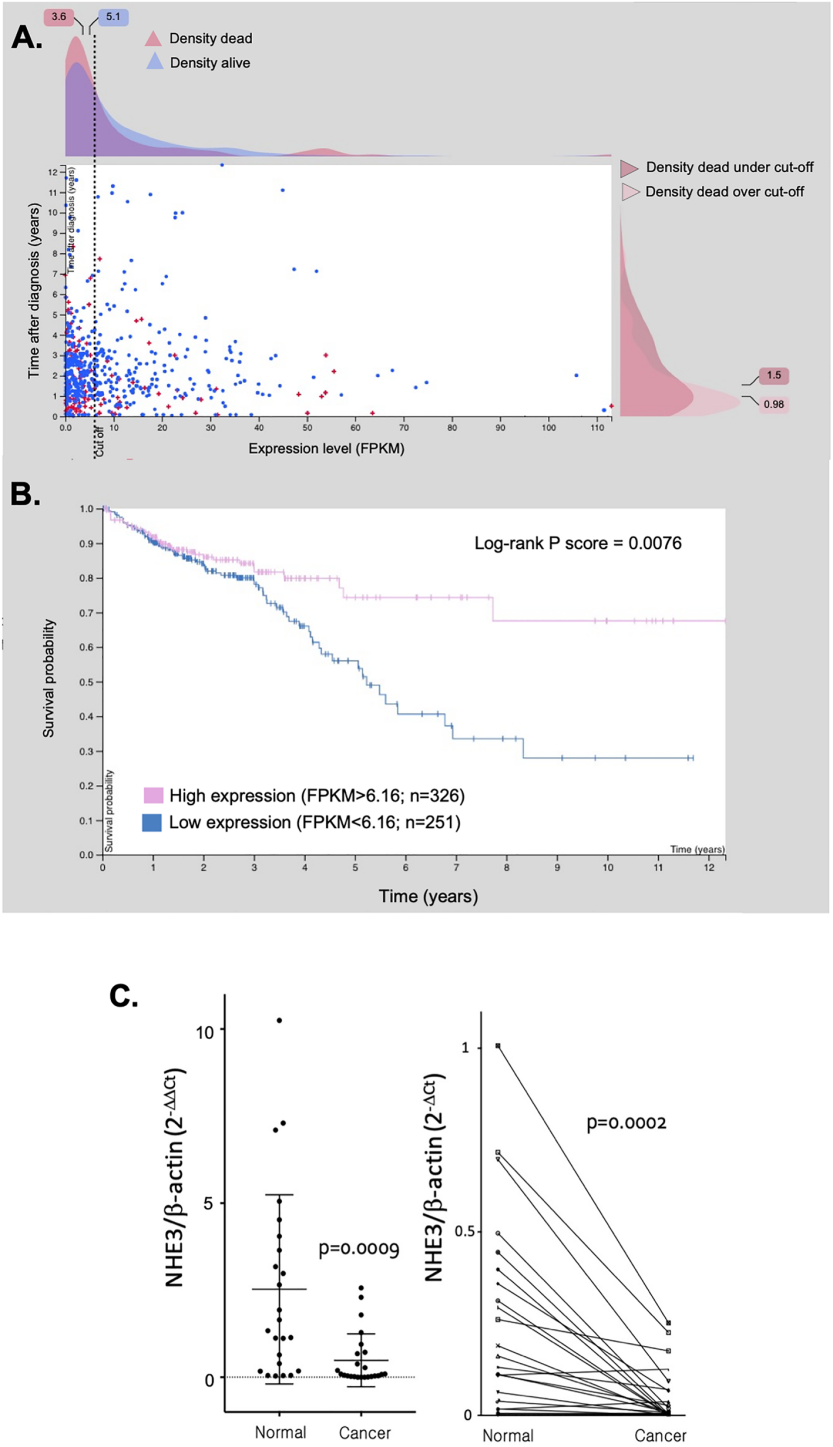
NHE3 mRNA expression in human colorectal cancer patients. (**A**) The Survival Scatter plot shows the clinical status (i.e., dead or alive) for all individuals in the patient cohort, based on the same data that underlies the corresponding Kaplan–Meier plot in (**B**). Patients that are alive at last time for follow-up are shown in blue and patients who have died during the study are shown in red. The X-axis shows the expression levels (FPKM; Fragments Per Kilobase of transcript per Million mapped reads) of the SLC9A3/NHE3 gene in the tumor tissue at the time of diagnosis. The Y-axis shows the follow-up time after diagnosis (years). Both axes are complimented with kernel density curves demonstrating the data density over the axes. The top density plot shows the expression levels (FPKM) distribution among dead (red) and alive patients (blue). The right density plot shows the data density of the survived years of dead patients with high and low expression levels respectively, stratified using the cutoff indicated by the vertical dashed line. (**B**) Kaplan–Meier plot of survival of CRC patients stratified by SLC9A3/NHE3 mRNA expression into "low expressors" (under cut off) or "high expressors" (over cut off). X-axis shows time for survival (years) and Y-axis shows the probability of survival, where 1.0 corresponds to 100%. Cut-off value for (**A**) and (**B**) were automatically defined as 6.16 to minimize the p-score. Data in (**A**,**B**) from https://www.proteinatlas.org/ENSG00000066230-SLC9A3/pathology/colorectal+cancer. (**C**) Validation of SLC9A3/NHE3 mRNA expression in an independent cohort of CRC patients. qRT-PCR was used to analyze NHE3 and β-actin mRNA expression using RNA isolated from 24 matched colonic biopsy pairs (tumor and unaffected tissue margin; 48 samples). The results of unpaired (left panel) and paired (right panel) analysis of NHE3 mRNA expression is shown. (Student t-test, n = 24; p value indicated in graph; GraphPad Prism 9, https://www.graphpad.com/).

We first utilized publicly available database generated by the TCGA Research Network: https://www.cancer.gov/tcga and The Cancer Genome Atlas (TCGA) and available through The Human Protein Atlas (proteinatlas.org) to test if NHE3 mRNA expression is associated with human CRC and patient survival. The surveyed results are derived from RNAseq data from biopsies taken at the time of diagnosis from 597 patients. This analysis can be reproduced and viewed at https://www.proteinatlas.org/ENSG00000066230-SLC9A3/pathology/colorectal+cancer. NHE3 mRNA expression did not correlate with the cancer stage (Supplementary Fig. S1) which suggests that it is downregulated early during the progression and cannot be considered as prognostic in CRC. However, survival analysis with a median follow-up time of 1.84 years indicated that NHE3 expression was associated with patient survival rate, with “low expressors” having significantly lower probability of 5-year survival (55%) compared to “high expressors” (70%) (Fig. 1A,B). To confirm decreased NHE3 expression in an independent cohort of CRC patients, we tested paired biopsy samples from unaffected colon tissues and colorectal cancer for expression of NHE3 using an array plate with normalized cDNA from 24 matched biopsy samples from human colon cancer and unaffected sites, covering 24 normal, 5 stage I, 5 stage IIA, 2 stage IIB, 2 stage IIIA, 3 stage IIIB, 2 stage IIIC, 1 stage III, and 4 stage IV samples (OriGene). Real-time qRT-PCR for human NHE3 transcript expression showed that in an independent comparison, NHE3 expression was significantly reduced in cancer biopsies (Fig. 1C, left panel; p = 0.0009). Similar effect was observed in paired comparison between unaffected regions and cancer (Fig. 1C, right panel; p = 0.0002), with 83% (20 out of 24) tested patients showing decreased NHE3 expression in cancer biopsies as compared to a non-affected region.

### NHE3 deficiency exacerbates tumor formation in Apc^Min/+^ mice

**Figure 2:**
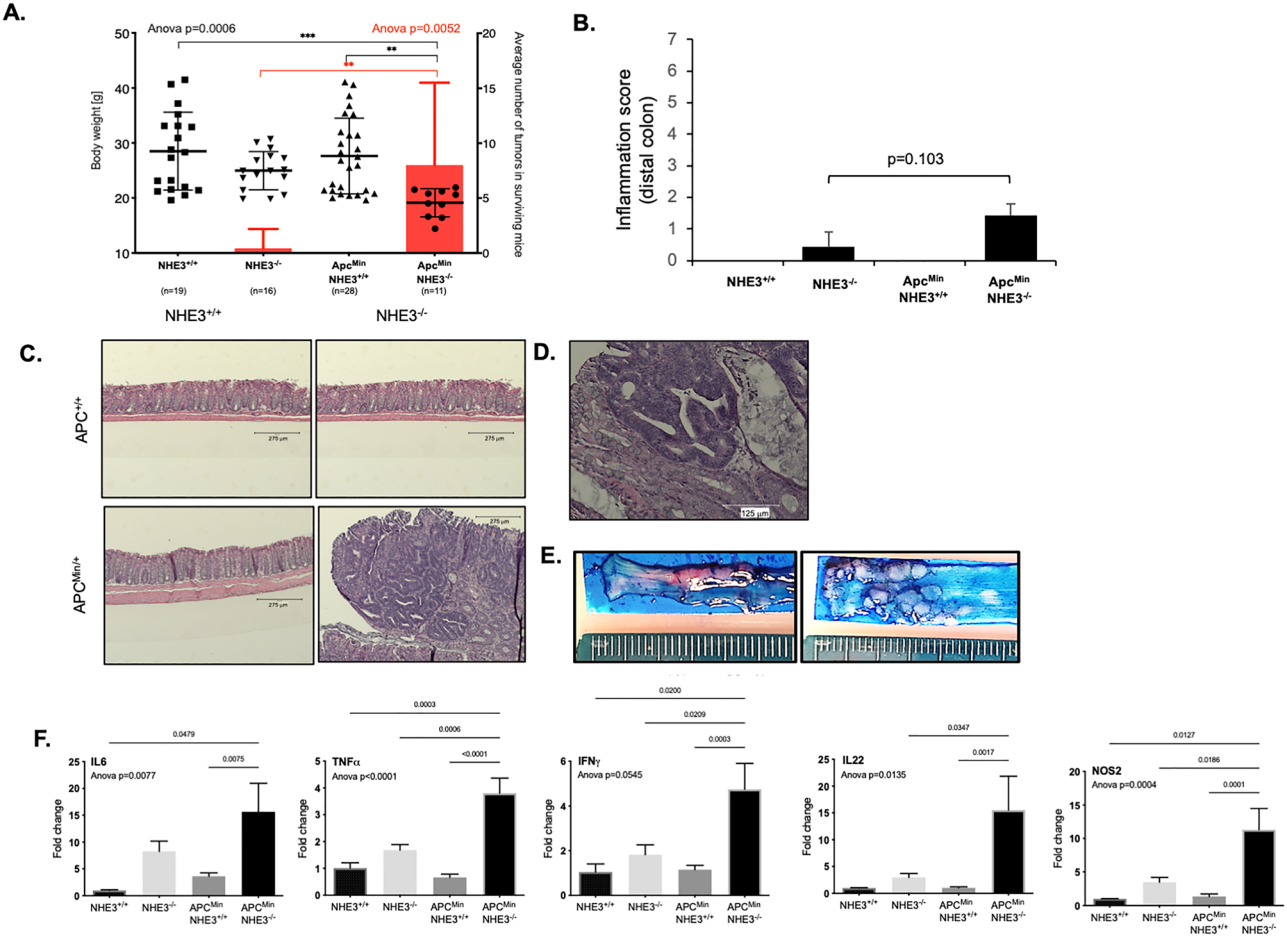
NHE3 expression decreases in human colorectal cancer and increased number of tumors in Apc^Min/+^NHE3^−/−^ mice is associated with lower body weight and increased colonic expression of proinflammatory cytokines. (**A**) Body weights (left Y axis, black symbols) and average number of polyps in surviving mice (right Y axis, red bars). Black symbols represent individual mice with median value marked with bold line; whiskers depict standard deviation. Red boxplots represent the mean values and standard deviations for the number of tumors per mouse. Statistical significance (in black for body weight and in red for the average number of tumors) was calculated using one-way ANOVA followed by Holm-Sidak’s multiple comparison test. (**B**) Bar graph summary of histological colitis scoring (reported p-value from Mann–Whitney test). (**C**) Representative H&E images of the distal colonic mucosa in the four genotypes. (**D**). High magnification representative image of tumor histology in Apc^Min/+^NHE3^−/−^ mice. (**E**) Representative image of methylene blue-stained distal colon with healthy mucosa in Apc^Min/+^NHE3^+/+^ mice and multiple adenomas in Apc^Min/+^NHE3^−/−^ mice. (**F**) Cytokine expression profile in the distal colon measured by qRT-PCR relative to GAPDH as a housekeeping gene. Statistical differences analyzed with one-way ANOVA followed by Tukey multiple comparison test.

Loss of NHE3 in mice is associated with chronic inflammation and with gut microbial dysbiosis^14,15,18,20,30^. Colitis developed in NHE3^−/−^ mice is microbiota-dependent and rederivation into a barrier facility (utilized in this study) dramatically reduces mucosal inflammation in this strain^20^. To investigate whether loss of NHE3 affects CRC development, we crossed NHE3^−/−^ mice onto Apc^Min/+^ background. We have generated 136 mice in the F2 generation. Due to an increased mortality observed in Apc^Min/+^NHE3^−/−^ and Apc^+/+^NHE3^−/−^ mice under 18 weeks of age (56% and 27%, respectively), 116 mice remained available for the study. NHE3^+/−^ mice, regardless of the Apc status, displayed a phenotype indistinguishable from NHE3^+/+^ littermates (data not shown), and were disregarded in further analyses. Overall, we analyzed 74 mice: 39 on Apc^Min/+^ background (20 females and 19 males) and 35 on Apc^+/+^ background (20 females and 15 males), with an average age 15.7 weeks and 17.1 weeks respectively (Table 1). Within this relatively early time frame, and in the microbial environment of a barrier facility where the mice where housed, and consistent with our prior work, Apc^+/+^NHE3^−/−^ mice did not present with overt histopathological colitis as evaluated by an unbiased pathologist and exemplified in Fig. 2B,C. Minor increases in mucosal cytokine (IL6, TNF, IFNg, IL22) and NOS2 mRNA expression were not statistically significant (Fig. 2C). Compared to other genotypes, mice in the Apc^Min/+^NHE3^−/−^ group had significantly lower body weight (Table 1, Fig. 2A). Colorectal adenomas were observed in all 4 out of 5 Apc^Min/+^NHE3^−/−^ males and in 4 out of 6 Apc^Min/+^NHE3^−/−^ females. Tumor multiplicity was 12 ± 7.3 and 10 ± 6.7 males and females, respectively, with no statistical significance between sexes (Table 1, Fig. 2).

**Table 1:** Number of mice in the study, their age, body weight, the number of adenomas and tumor multiplicity for each genotype. Statistical differences between genotypes for body weight and tumor multiplicity were analyzed with one-way ANOVA test followed by Tukey’s multiple comparison test.

	Mice (n)			Age (weeks)				Body weight (g)				Mice with adenomas (n)				Mean tumor multiplicity			
	All	Females	Males	All		Females	Males	All		Females	Males	All		Females	Males	All		Females	Males
Apc^Min/+^NHE3^−/−^	11	6	5	15.1 ± 1.4		15.4 ± 0.4	14.7 ± 2.1	19.12 ± 2.6^a^		19.1 ± 2.5	19.1 ± 3.0^a^	8		4	4	8.0 ± 7.5^a^		6.7 ± 7.3^a^	9.6 ± 8.3^a^
Apc^Min/+^NHE3^+/+^	28	14	14	16.3 ± 1.8		16.3 ± 2.1	17.1 ± 1.5	27.6 ± 6.9^b^		23.5 ± 5.9	32.1 ± 4.8^b^	0		0	0	0^b^		0^b^	0^b^
Apc^+/+^NHE3^−/−^	16	8	8	16.6 ± 1.7		17.1 ± 2.2	16.0 ± 0.7	25.0 ± 3.5^ab^		22.3 ± 2.1	27.7 ± 2.2^c^	1		1	0	0.4 ± 1.8^b^		0.9 ± 2.5^b^	0^b^
Apc^+/+^NHE3^+/+^	19	12	7	17.7 ± 2.4		17.3 ± 2.4	18.3 ± 2.4	28.5 ± 7.1^b^		24.2 ± 4.2	35.9 ± 4.0^d^	0		0	0	0^b^		0^b^	0^b^
ANOVA p value								0.0006		0.1473	< 0.0001					< 0.0001		0.001	< 0.0001

### Increased tumor burden in Apc^Min/+^NHE3^−/−^ mice is associated with increased mucosal inflammation and DNA damage in the surface epithelium

We did not observe any macroscopic lesions in the small intestine of Apc^Min/+^ mice regardless of NHE3 status (not shown). Tumors observed in the Apc^Min/+^NHE3^−/−^ mice were restricted to the distal colon, usually localized within 2 cm from rectum. qRT-PCR analysis of mucosal expression of selected inflammatory mediators in Apc^Min/+^NHE3^+/+^ mice did not indicate active inflammation, while Apc^+/+^NHE3^−/−^ mice developed very mild or no inflammation consistent with our previous report^20^ (Fig. 2B,C,F). These changes were reflected in histological analysis of H&E-stained tissues (Fig. 2C) which indicated mild mucosal inflammation as assessed by an experienced pathologist blinded to the study design based on lesion scoring criteria for mouse intestinal lesions modified from Burich et al.^31^ (distal colon segment score 0.43 ± 0.46).

Histologic evaluation of distal colons of Apc^Min/+^ NHE3^−/−^ mice revealed a predominance of adenomas with high-grade dysplasia (characterized by pronounced nuclear atypia, loss of epithelial cell nuclear polarity, mucosal gland cribriform appearance), with fewer lesions characterized as adenomas with low-grade dysplasia (characterized by elongated and crowded crypt cells with hyperchromatic nuclei that maintain polarity with respect to the basement membrane) or intraepithelial neoplasia (carcinoma in situ) defined as dyplastic crypt foci < 0.5 mm diameter^32^. Mild mixed mucosal inflammation and crypts filled with necrotic debris were associated with the adenomas. These lesions were not observed in the groups of Apc^Min/+^, NHE3^−/−^, or wildtype mice. The abnormal morphology with multiple adenomas was limited to a distal colon (Fig. 2B–E). Apc^Min/+^NHE3^−/−^ mice had significantly higher expression of IL-6, TNFα, IFNγ, IL-22, and NOS2 in the distal colonic mucosa compared with other genotypes (Fig. 2F).

**Figure 3:**
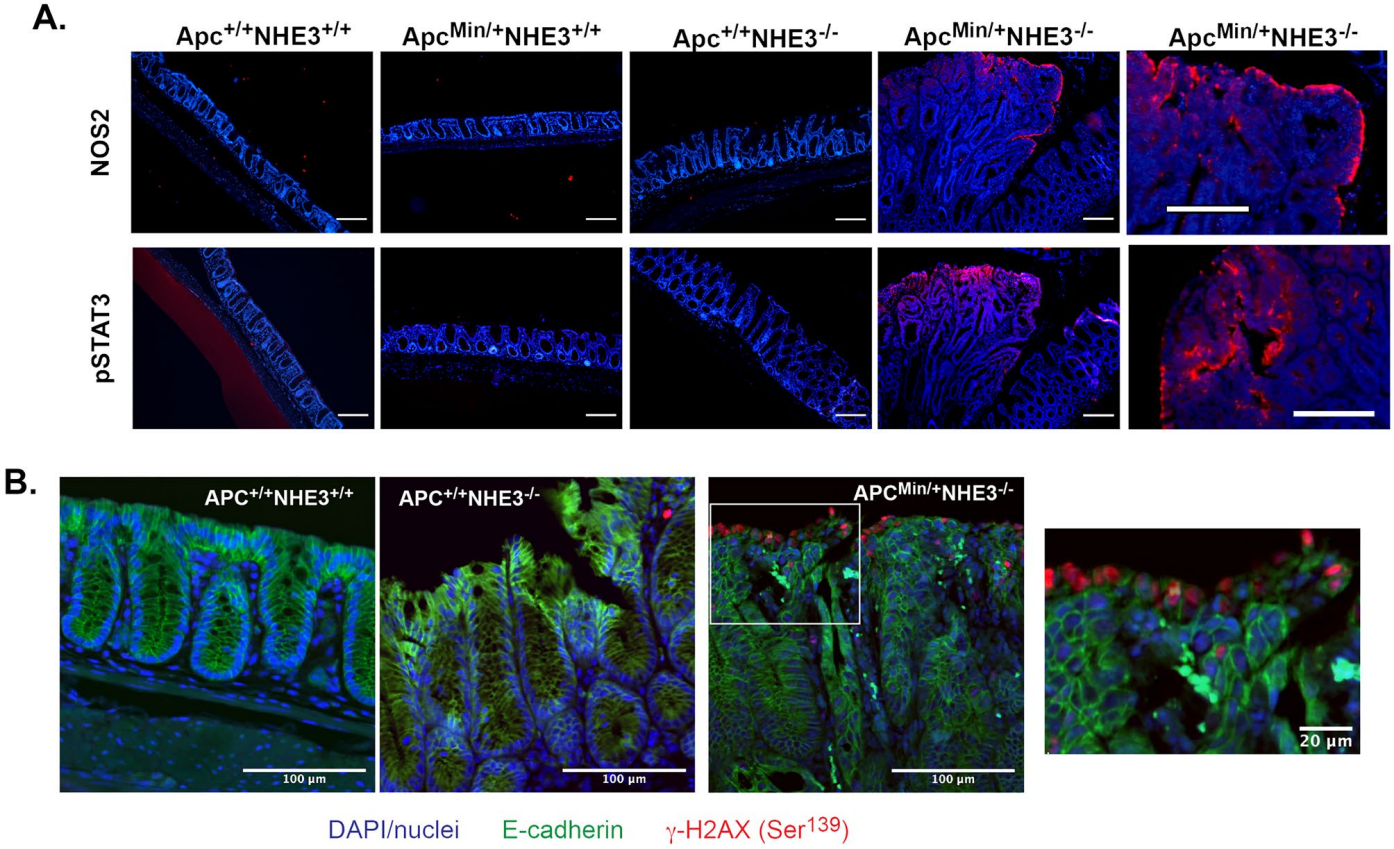
Tumor surface epithelia in Apc^Min/+^NHE3^−/−^ mice show increased level of NOS2, activation of STAT3, and DNA damage. (**A**) Representative immunofluorescence (IF) staining for NOS2 and pSTAT3 (both in red), with blue DAPI nuclear counterstaining in all four genotypes. The white scale bars represent 200 μm. High magnification images of NOS2 and pSTAT3 IF from Apc^Min/+^NHE3^−/−^ mice are in the rightmost two panels. (**B**) DNA damage (double stranded DNA breaks) was evaluated by immunofluorescent staining for phosphorylated histone H2AX (γ-H2AX). Tissue specimens were stained for γ-H2AX (red), E-cadherin (green), and nuclei were counterstained with DAPI (blue). The scale bars represent 100 mm or 20 mm for the higher magnification insert.

Increased expression of NOS2 and IL-22 in Apc^Min/+^NHE3^−/−^ was consistent with increased immunofluorescent staining for NOS2 and pSTAT3, the latter as a non-specific marker of downstream IL-22 signaling known to be activated in the development of human colon cancer^33^ (Fig. 3A). The staining was mostly localized on the luminal surface of adenomas, where the highest expression of NHE3 would be normally expected. IL-22 interacts with nitric oxide, a product of NOS2 activity, to promote DNA damage in a model of *Helicobacter hepaticus*-induced colon cancer^34^. We therefore tested the level of histone γ-H2AX phosphorylated at Ser^139^ as a sensitive marker of DNA double stranded breaks (DSBs). The immunofluorescence staining for γ-H2AX was dramatically increased in the tumor surface in Apc^Min/+^NHE3^−/−^ mice, with no discernable signal in other genotypes (Fig. 3B).

### Microbial community structure in the Apc^Min/+^NHE3^−/−^ mice

**Figure 4:**
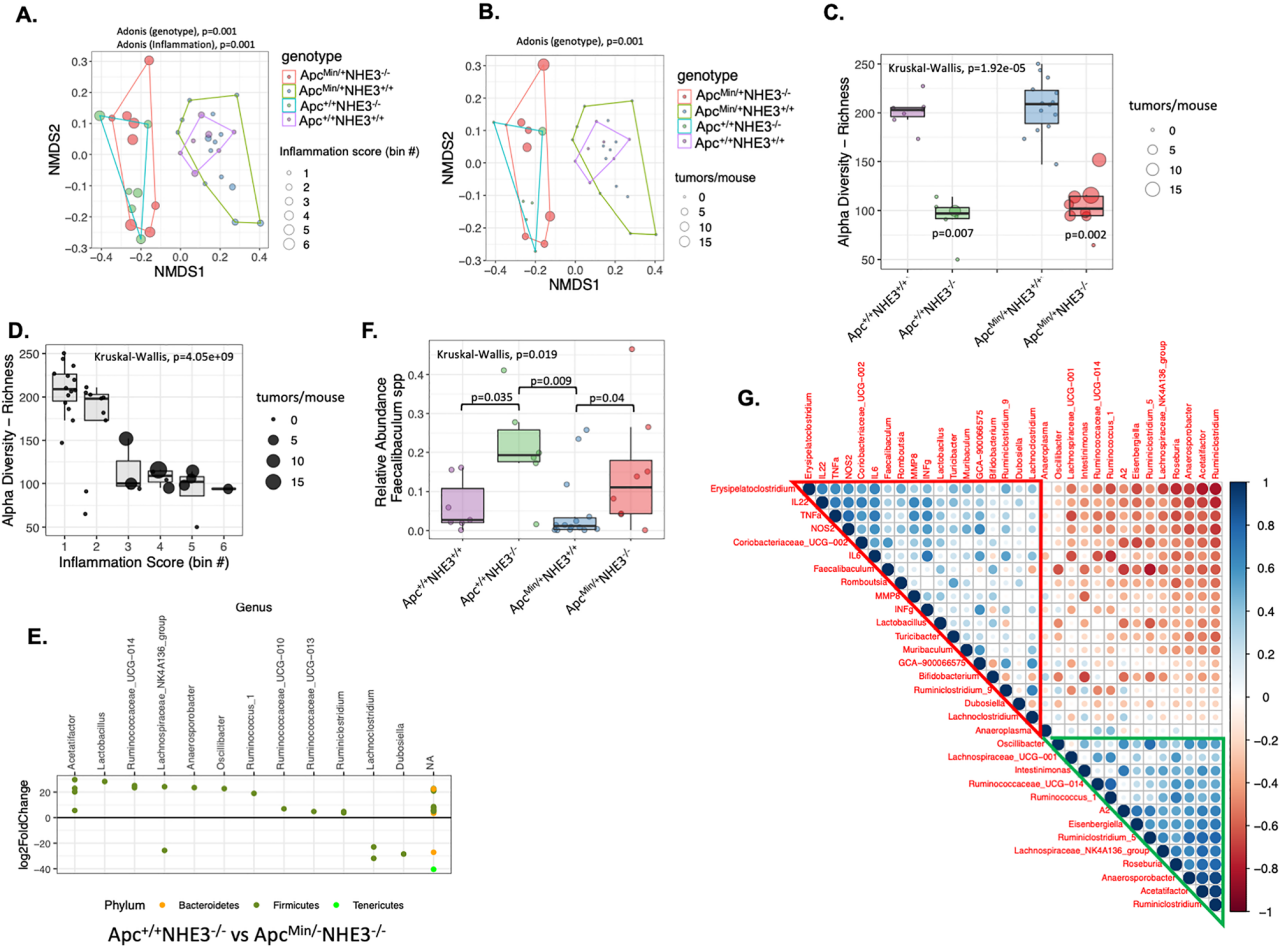
NHE3 deficiency leads to microbial dysbiosis and correlates with severity of inflammation and tumor number. Non-metric Multidimensional Scaling (NMDS) analysis based on Bray–Curtis distances (**A**,**B**), and richness (a-diversity measure; **C**,**D**) were used to analyze the groups in the context of genotype, inflammation score, and tumor number. In all panels, dots represent samples from individual mice. The size of the dots corresponds to the inflammation score (**A**) or to the tumor multiplicity (**B**–**D**). Polygons connect points within the same cluster representing individual genotypes (**A**,**B**). In the bar plots (**C**,**D**) the lower and upper hinges represent the first and third quartiles respectively. The whiskers extend to the largest and lowest values. The middle line represents the median value. Permutational Multivariate Analysis of Variance Using Distance Matrices (Adonis test, **A**,**B**) or Kruskal–Wallis H test followed by Wilcoxon rank sum test with continuity correction (panels **C**,**D**) were used. Bonferroni p value adjustment method was used. (**E**) The impact of APC status on fecal microbial composition in NHE3^−/−^ mice was analyzed using DESeq2, differential abundance analysis tool. Each dot represents an individual ASV. The color of the dots represents a specific phylum (yellow—Bacteroidetes, dark green—Firmicutes, bright green Tenericutes). For this presentation, only significantly different taxa (Wald significance test < 0.05) with at least twofold change (absolute value of log2FoldChange > 1) were selected. (**F**). Relative abundance of *Faecalibaculum* spp. in fecal samples in all analyzed genotypes. Dots represent individual samples/mice, lower and upper hinges of the bars represent the first and third quartiles respectively. The whiskers extend to the largest and lowest values. The middle line represents the median value. Statistical differences were analyzed using Kruskal–Wallis H test followed by Wilcoxon rank sum test with continuity correction. (**G**) Heatmap of Spearman’s correlation between relative abundances of fecal microbial general (for the core gut genera, which summarized relative abundance across all samples for individual genus as higher than 50%). The circle size reflects the absolute value of corresponding correlation coefficient. Red and blue color represent positive and negative correlation respectively. Darker color represents stronger correlations. The green triangle highlights potentially beneficial bacteria negatively correlated with proinflammatory cytokines and potential pathobionts. The red triangle highlights genera positively correlated with inflammation indices. The heatmap was generated with corplot R package (ver 0.92; https://github.com/taiyun/corrplot).

Stool samples collected from all four genotypes (74 mice, Table 1) were sequenced and analyzed. The number of adenomas in the colon, cytokine levels, and inflammation scores were available for 35 mice (7 for Apc^+/+^NHE3^+/+^, 6 for Apc^+/+^NHE3^−/−^, 14 for Apc^Min/+^NHE3^+/+^, and 8 for Apc^Min/+^NHE3^−/−^). Therefore, all further microbial analysis was limited to those 35 mice. Bray–Curtis dissimilarity based non-metric multidimensional scaling (NMDS) analysis showed that both NHE3^+/+^ cohorts clustered separately from the NHE3^−/−^ and Apc genotype did not further compound the effect of NHE3 deletion (Fig. 4A,B). Changes in the microbial community due to NHE3 deficiency were associated with an increased inflammation score (Fig. 4A); however, only in Apc^Min/+^NHE3^−/−^ mice, microbiota was associated with the increased number of tumors (Fig. 4B). Permutational Multivariate Analysis of Variance Using Distance Matrices (PERMANOVA, *vegan::adonis2*) showed significant microbial community differences for genotype (p = 0.001, R^2^ = 0.33541, F = 5.215), inflammation score (p = 0.001, R^2^ = 0.20669, F = 8.5977), number of tumors (p = 0.003, R^2^ = 0.09, F = 3.4081), and body weight (p = 0.002, R^2^ = 0.11057, F = 4.1023). No statistical differences were observed when the test was performed for cage effect (p = 0.359, R^2^ = 0.03078, F = 1.0479, Supplementary Fig. S2A) and for sex differences (p = 0.202, R^2^ = 0.04034, F = 1.3871, Supplementary Fig. S2B). NHE3 status significantly (Kruskal–Wallis test, p < 0.001) impacted the alpha diversity (ASV richness; ASV—amplicon sequence variant) (Fig. 4C). All NHE3^−/−^ mice, regardless of Apc status, had significantly lower richness. In Apc^Min/+^NHE3^−/−^ mice, tumor multiplicity correlated with increasing degree of inflammation (Fig. 4D).

Differential abundance analysis on genus level using DeSeq2 package showed that genera *Enterococcus*, *Candidatus*_*Stoquefichus*, *Erysipelatoclostridium*, *Marvinbryantia*, and *Ruminiclostridium_9* from the phylum *Firmicutes* were significantly increased in NHE3 deficient mice comparing to NHE3^+/+^ mice (both on Apc^+/+^ background), whereas genera *Eisenbergiella*, Oscillibacter, *Ruminococcaceae*_UCG—013, *Harryflintia*, GCA—900066575, *Ruminiclostridium*, *Ruminiclostridium*_6, *Ruminococcaceae* (UCG_003 and UCG_010), *Lachnospiraceae* (UCG_006, FC020_group, and UCG_001), A2, *Clostridium_sensu_stricto*_1, *Acetatifactor*, UBA1819, *Ruminococcus*_1, and *Anaerosporobacter* showed significantly decrease abundance (Fig. S3A). Several genera were assigned to more than one ASV (e.g. *Lachnospiraceae*_NK4A136_group, *Roseburia*, *Ruminococcaceae*_UCG—014, *Ruminiclostridium*_5), however majority of the ASVs showed decreased abundance in NHE3 deficient mice, except for the genus *Anaerotruncus* and *Lachnoclostridium* for which two out of three ASVs increased in NHE3^−/−^ mice (Supplementary Fig. S3A). We also observed a significant increase in the genus *Enterorhabdus* form the phylum *Actinobacteria* and *Muribaculum* from the phylum *Bacteroidetes*. A bile tolerant, sulphate reducing bacteria from the genus *Bilophila*, phylum *Proteobacteria*, were significantly decreased in NHE3^−/−^ mice. Taxonomic composition analysis in fecal samples from all four genotypes showed that NHE3 deficiency was a dominant factor modulating gut microbiome, and both NHE3-deficient genotypes appeared to be similar (Supplementary Fig. S3B). Nevertheless, DESeq2 differential abundance comparison of Apc^+/+^NHE3^−/−^ and Apc^Min/+^NHE3^−/−^ mice identified several significantly different taxa (Fig. 4E). In this case, introduction of the Apc^Min^ allele was associated with increased relative abundance of *Acetatifactor*, *Lactobacillus*, *Ruminococcaceae UCG-014*,* Lachnospiraceae*_NK4A136_group, *Anaerosporobacter*, *Oscillibacter*, *Riminococcus*_1, *Ruminococcaceae_UCG013*, and* Ruminiclostridium*. Only *Lachnoclostridium* and *Dubosiella* showed significant decrease (Fig. 4E). *Faecalibaculum* genus with only one species, *F. rodentium*, which was recently reported to reduce tumor growth in Apc^Min/+^ model^35^, showed increasing tendency in our Apc^Min/+^ mice, regardless of the NHE3 status (Fig. 4F), though without reaching statistical difference.

Spearman's rank correlation coefficient analysis (Fig. 4G) showed high correlation between selected genera and the tested proinflammatory cytokines. *Erysipelatoclostridium* was highly positively correlated with increased IL-22, TNFα, NOS2, and IL-6 and negatively correlated with *Ruminiclostridium, Acetatifactor*, *Anaerosporobacter, Roseburia, Eisenbergiella* and other genera involved in SCFA metabolism. IL-6 and INFγ were positively correlated with *Coriobacteriaceaea*_UCG-002 and GCA-900066575. To a lesser extent, the selected inflammatory mediators were positively correlated with genus *Romboutsia*, *Lactobacillus*, *Turicibacter* and *Muribaculum* (Fig. 4G, red triangle). All genera considered as SCFA producers with a reported positive impact on the gut health were positively correlated to each other and clustered together (Fig. 4G, green triangle).

We also compared mucosal adherent microbiome from tumors and adjacent colonic mucosa in Apc^Min/+^NHE3^−/−^ mice and found no statistically significant difference in alpha and beta diversity (richness and Bray–Curtis distance matrices; Supplementary Fig. S4A,B). Although at some tumor sites we observed a relative expansion of the *Escherichia/Shigella* genus (in 5/20 samples), none of the genera reached statistical significance in DeSeq2 analysis (Supplementary Fig. S4C). This observation suggested that the effects of NHE3 deficiency that affected the entire epithelium dominated any potential localized effects at the tumor site. Nevertheless, in paired Wilcoxon signed-rank test, we identified four genera with relative abundance statistically lower in the tumor mucosa, compared to unaffected mucosa in Apc^Min/+^NHE3^−/−^ mice: *Ruminiclostridium_5, Muribaculum, Erisipelatoclostridium,* and *Alistipes* (Supplementary Fig. S4D).

### Functional prediction of the compositional changes of fecal microbiota

We employed PICRUSt2 algorithm^36^, a microbiome functional prediction tool based on the 16S rRNA marker gene. MetaCyc (MetaCyc Metabolic Pathway Database)^37^ pathway abundance was calculated through structured mapping of EC gene families to pathways and differential abundance of pathways was calculated using DeSeq2 package. When comparing NHE3^−/−^ to NHE3^+/+^ mice on Apc^+/+^ background (Supplementary Fig. S5A) or Apc^Min/+^NHE3^−/−^ to WT controls (Supplementary Fig. S5B), we observed a dramatically and significantly increased abundance of bacteria with active glyoxylate cycle. This alternative shortened TCA cycle (glyoxylate bypass) enables microorganisms to use substrates that enter central carbon metabolism at the level of acetyl-CoA, such as fatty acids, alcohols, and esters. Similarly, pathways involved in peptidoglycan biosynthesis, a polymer forming bacterial outer cell wall, were increased. Several superpathways of menaquinol syntesis were significantly increased. Menaquinols, reduced forms of menaquinones (natural form of vitamin K2), are linked to nitrite reductases as electron donor and are involved in the detoxification of NO in many enterobacterial species^38^. Superpathway of sulfate assimilation and cysteine biosynthesis, sulfate reduction I, ADP-l-glycero-β-d-manno-heptose biosynthesis, isopropanol biosynthesis, and glucose and glucose-1-phosphate degradation pathway abundances were decreased in Apc^Min/+^NHE3^−/−^ mice (Supplementary Fig. S5B).

### Microbiota-independent consequences of NHE3 knockdown in colorectal cancer cells

**Figure 5:**
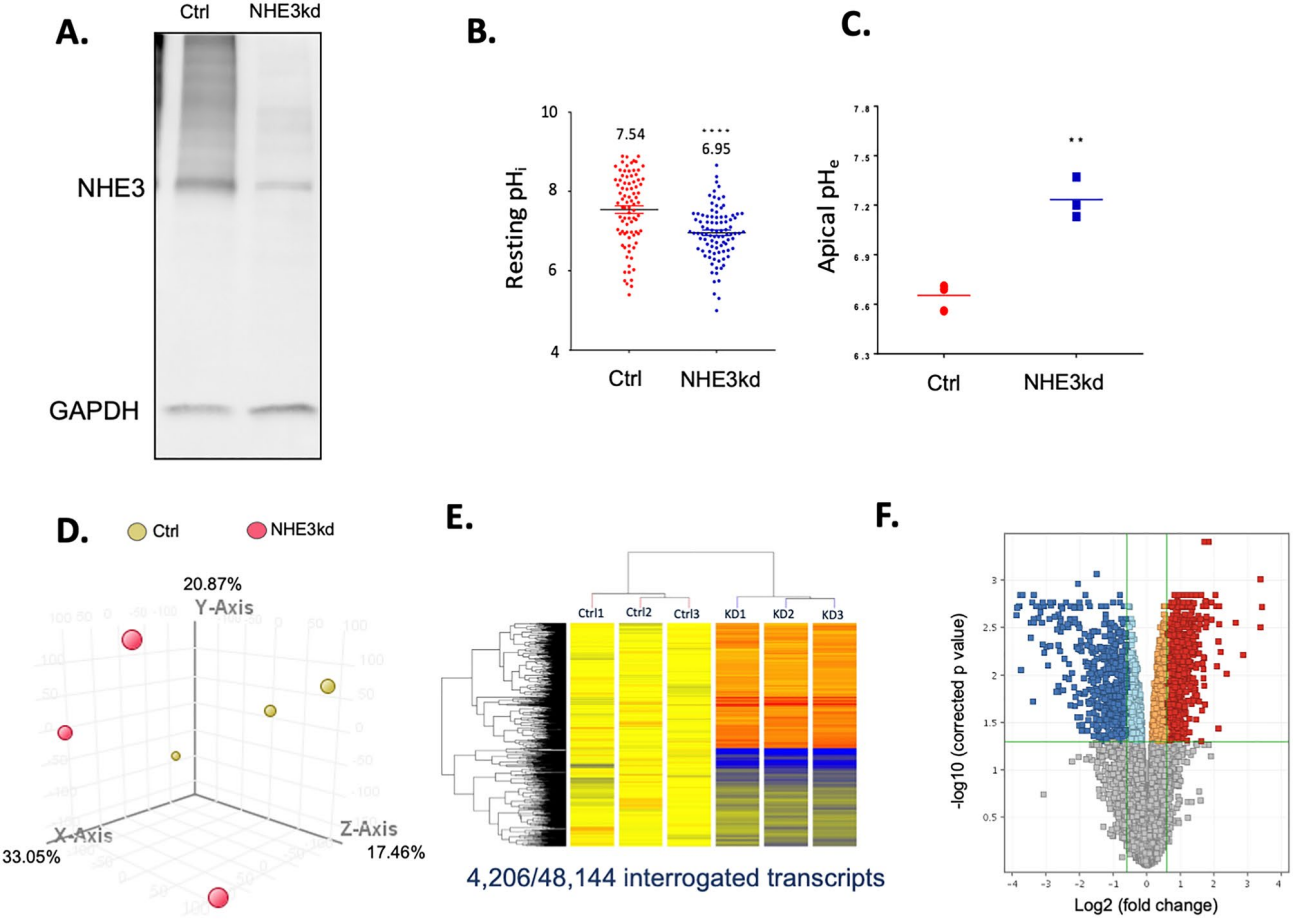
The effects of NHE3 knockdown in SK-CO15 colonic adenocarcinoma cells on cellular pH and gene expression profile. (**A**) Representative Western blot of NHE3 protein expression in control (Ctrl) or NHE3-knockdown cells (NHE3kd) stably transduced with lentivirus with scrambled or NHE3-specific shRNA. Full blot shown in Supplementary Fig. S7. (**B**) Decreased resting intracellular pH (pHi) in NHE3kd cells (Student t-test, ****p < 0.0001). (**C**) Increased extracellular apical pH (pHe) in NHE3kd cells grown as monolayers in Transwells (Student t-test, **p < 0.01). Differences in global gene expression profile of Ctrl and NHE3kd cells determined by microarray analysis: (**D**) Principal component analysis of samples without filtering; (**E**) heat map of differentially expressed 48,144 transcripts; (**F**) Volcano plot of differentially expressed genes in Ctrl vs. NHE3kd cells. (**D**–**F**) generated by GeneSpring GX software (https://www.agilent.com/en/product/software-informatics/genomics-software-informatics/gene-expression/genespring-gx).

To address whether loss of NHE3 in transformed colon epithelial cells affects the cell sensitivity to DNA damage independently of the luminal bacteria, we utilized SK-CO15 human colon adenocarcinoma cells. These cells are relatively unique among CRC cell lines in that they express functional NHE3^39^ thus providing a suitable model to study the phenotypic consequences of NHE3 downregulation. We identified only one missense variant in KRAS gene (c.35G > A; pGly12Asp; variant frequency 0.963) in SK-CO15 cells, and no mutations in TP53, PIK3CA, BRAF, or EGFR genes, consistent with late adenoma genotype. For comparison, T84 cells, which do not express NHE3 had both KRAS c.38G > A (p.Gly13Asp) missense variant (variant frequency 0.604) and TP53 c560-1G > T splice acceptor variant (frequency 0.995). Stable transduction with *SLC9A3* shRNA lentivirus resulted in ca. 76% decrease in total NHE3 protein, as compared to cells transduced with control (scrambled) shRNA (NHE3kd; Fig. 5A). Consistent with impaired NHE3 activity, NHE3kd cells showed significantly lower resting intracellular pH (pH_i_; Fig. 5B) and increased extracellular apical pH (pH_e_) when measured in cells grown as monolayers in Transwells (Fig. 5C). We performed transcriptional profiling of the control and NHE3kd SK-CO15 cells using microarray analysis and found a significant effect of NHE3 knockdown (Fig. 5D). Of all probe sets identified as differentially expressed (corr. P < 0.5; Fig. 5E), 2042 probe sets had a ≥ 1.5-fold change (Fig. 5F). Of those, gene ontology analysis identified multiple biological processes potentially impacted by the altered gene expression, and selected categories are depicted in Supplementary Table S1. These included cell division and replication, inflammatory response, response to DNA damage, transcriptional and post-transcriptional regulation, translational modifications, cell migration, and response to hypoxia.

**Figure 6:**
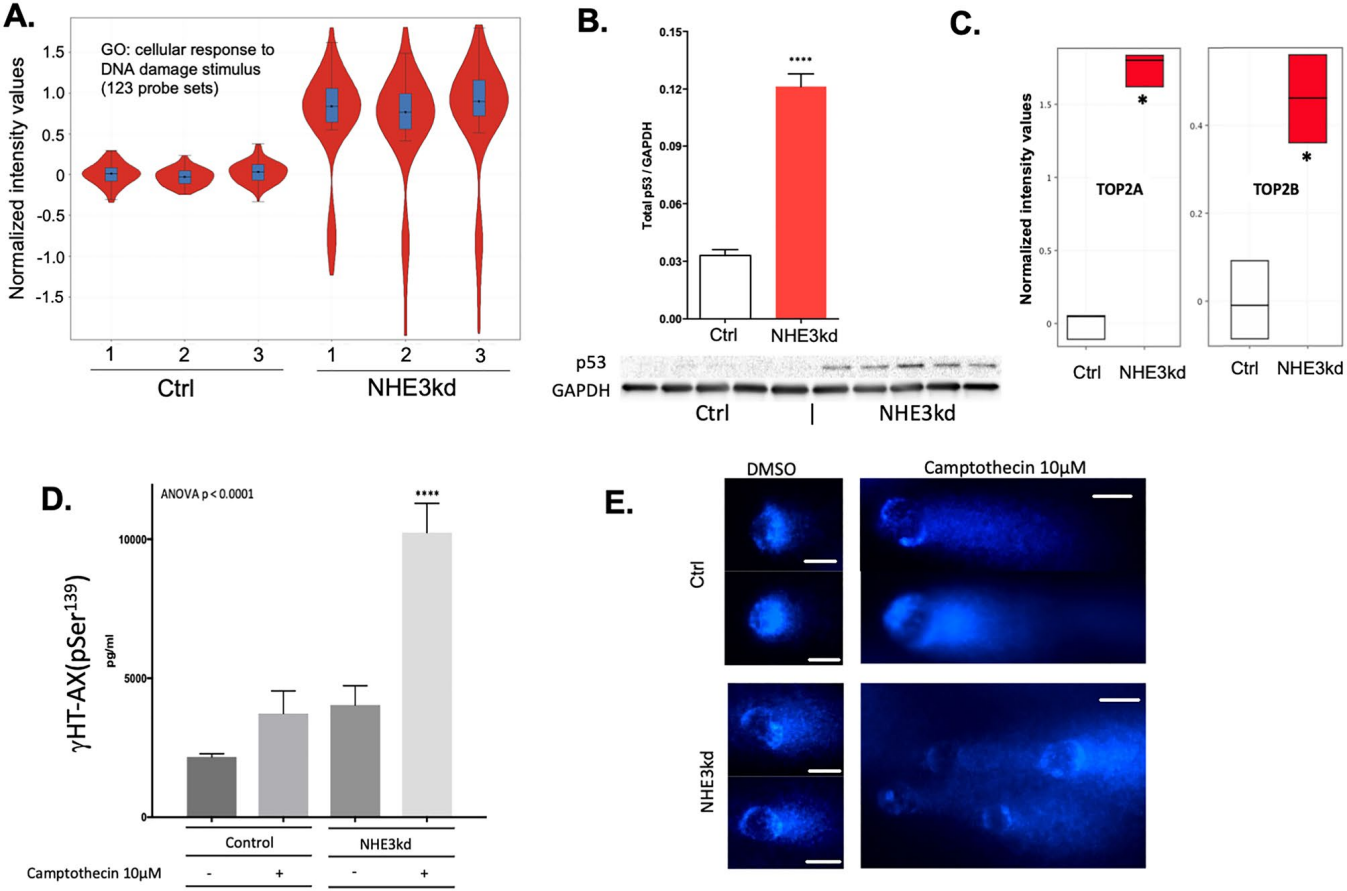
NHE3 knockdown in SK-CO15 colonic adenocarcinoma cells leads to enhanced susceptibility to DNA damage. (**A**) Gene set enrichment analysis (GSEA) of transcripts encoding genes from gene ontology category of cellular response to DNA damage stimulus. (**B**) Increased expression of wild-type p53 protein in NHE3kd SK-CO15 cells (Student t-test, ****p < 0.0001). (**C**) Increased expression of topoisomerase 2 isoforms in NHE3kd SK-CO15 cells (moderated T-test, *adj. p < 0.05). (**D**) ELISA analysis if γHT-AX histone in the lysates of Ctrl and NHE3kd SK-CO15 cells treated with and without 10 mM camptothecin. The results of ANOVA (p < 0.0001) are indicated. Asterisks depict a statistical difference between camptothecin-treated NHE3kd cells as compared to all other cells/treatments (Post hoc Fisher PLSD test; ****p < 0.0001). (**E**) High magnification of comet assay for cells and treatment as in (**D**). Lower magnification provided in Supplementary Fig. S5. White scale bars indicate 10 µm.

Driven by the gene ontology analysis, we focused on selected cellular events potentially regulated by NHE3 downregulation in colon cancer cells. Majority of genes associated with DNA damage were upregulated (Fig. 6A). Both microarray and qRT-PCR showed significantly increased expression of p53 (TP53) mRNA and protein in NHE3kd cells (Fig. 6B), consistent with the cellular response to DNA damage^40^. Reduced NHE3 expression was associated with significant increase in the expression of topoisomerase 2a and 2b (TOP2A/TOP2B) isoforms (Fig. 6C). TOP2 is known to be abnormally activated by acidic pH to potently induce DNA damage and cytotoxicity in tumor cells^41^. At baseline, γ-H2AX level in NHE3kd cells was modestly elevated, but NHE3kd cells were exquisitely sensitive to camptothecin-induced DNA damage as demonstrated by γ-H2AX ELISA and comet assay (Fig. 6D,E). A representative image of the comet assay at lower magnification is provided in Supplementary Fig. S6.

**Figure 7:**
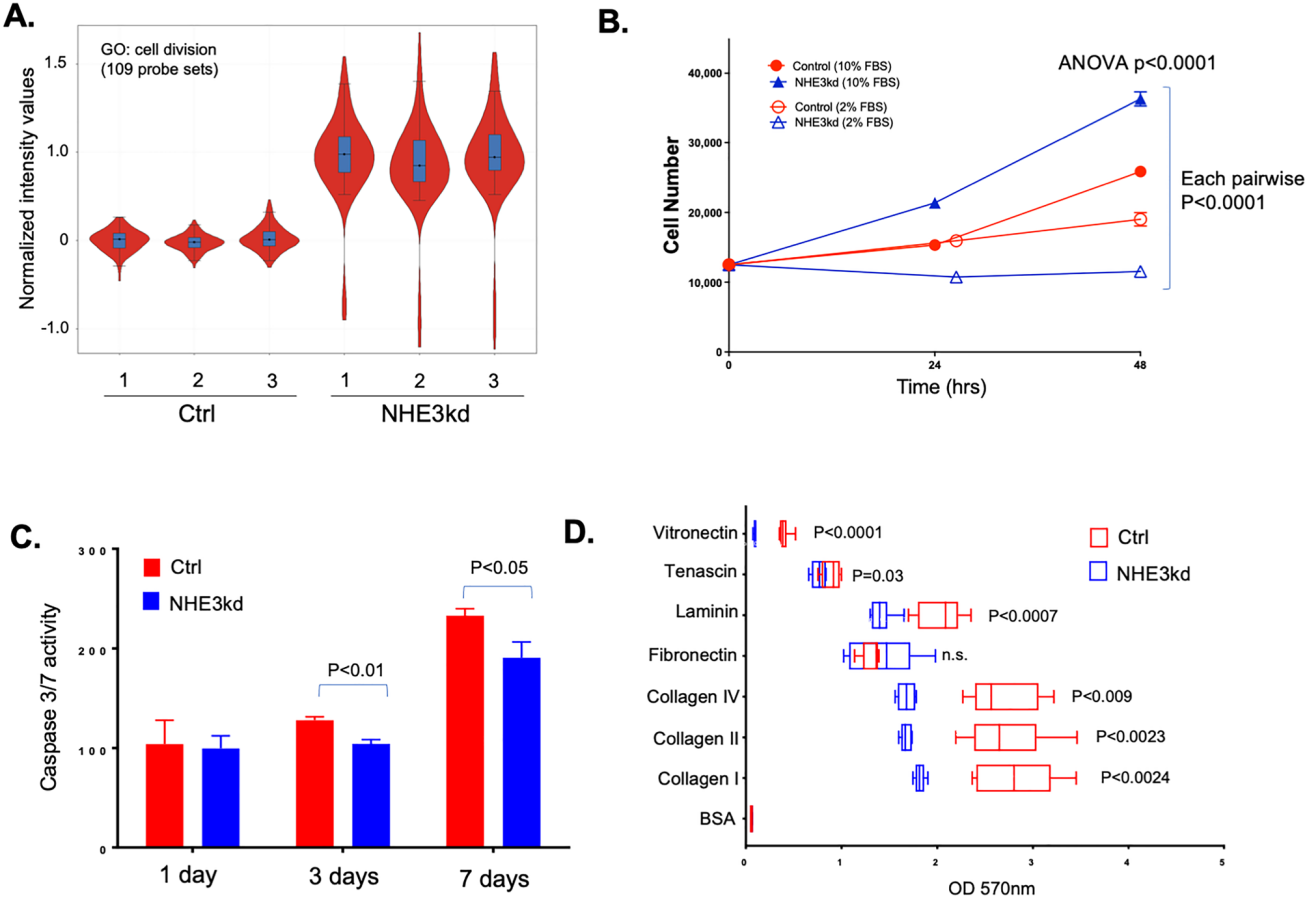
NHE3 knockdown in SK-CO15 colonic adenocarcinoma cells leads to increased proliferation, decreased apoptosis and decreased extracellular matrix (ECM) adhesion. (**A**) Gene set enrichment analysis (GSEA) of transcripts encoding genes from gene ontology category of cell division. (**B**) proliferation of Ctrl (red) and NHE3kd (blue) cells cultured with 2% (open symbols) or 10% (filled symbols) fetal bovine serum (FBS) in the media over 48-h period. The result of ANOVA test is indicated. In pairwise Fisher PLSD post-hoc analysis, all pairs of were significantly different at 48 h (p < 0.0001). (**C**) Caspase 3/7 activity assay as a measure of baseline apoptosis rate in Ctrl (red) and NHE3kd (blue) cells. (**D**) Differences in adhesion of Ctrl (red) and NHE3kd (blue) cells to an array of extracellular matrix proteins. P values indicate the result of unpaired T-test for each ECM protein, respectively.

Similarly, majority of genes associated with cell division were upregulated in NHE3kd cells (Fig. 7A). Consistent with this observation, proliferation rate of NHE3kd cells grown in 10% FBS was significantly higher, with doubling time reduced 2.5-fold compared to controls [27.2 vs. 49.5 h] (Fig. 7B). Interestingly, NHE3kd cells were more sensitive to growth factor deprivation (2% FBS), which inhibited NHE3kd cell proliferation entirely (Fig. 7B). Reduced NHE3 expression and activity in NHE3kd cells was also associated with reduced apoptosis at baseline, as demonstrated by caspase 3/7 activity assay (Fig. 7C). Since cell migration is associated with altered interaction with the extracellular matrix (ECM) and may implicate NHE3 in regulation of the metastatic potential of colon cancer cells, we tested the adhesion of control and NHE3kd cells to various ECM proteins. With the exception of fibronectin, NHE3kd cells showed significantly decreased adhesion to all other tested ECM substrates (Fig. 7D).

## Discussion

This is the first report conclusively demonstrating that colorectal cancer is associated with the loss of NHE3 expression and that this has functional consequence for tumor growth, mucosal inflammation, gut microbiota in Apc^Min/+^NHE3^−/−^ mice, and that NHE3 deficiency promotes the intrinsic proliferative, apoptotic, and adhesive properties of colorectal cancer cells. These observations are novel and provocative considering the prior focus of the cancer field on the basolateral proton extrusion by NHE1, which led to a generalized conclusion that inhibition of Na^+^/H^+^ exchange could lead to reduced tumor cell motility, invasion, proliferation, and growth. Combination of the results from human tumor biopsies, mouse and cancer cell models strongly suggests that local loss NHE3 may promote colonic tumor growth and consequently contribute to limiting patient survival.

To address the role of NHE3 deficiency in vivo, we utilized a well-established model of Apc haploinsufficiency. Apc^Min/+^ mice on C57BL/6 (BL6) and AKRxB6 backgrounds are known to develop both small intestinal and colonic tumors^23^. Moreover, additional treatment of Apc^Min/+^ mice with AOM or other carcinogens increases tumor malignancy and simultaneously shortens the time till tumor development^42–44^. Similarly, inflammation is known to accelerate colonic adenoma formation in this strain^25^. To reduce the over effect of inflammation developed by NHE3^−/−^ house when housed in a conventional facility, all experiments were performed in a barrier facility in the Bio5 vivarium at the University of Arizona, an environment we previously demonstrated as insufficient to lead to colitis in rederived NHE3^−/−^ mice^20^. In fact, as we demonstrated in Fig. 2, Apc^+/+^NHE3^−/−^ mice in this study did not show statistically significant differences in body weights or mucosal cytokine expression, and only very mild histological changes, consistent with this previous report. At 15 weeks, the age chosen to limit mortality associated with compound NHE3/Apc deficiency, F2 cross between B6 Apc^Min/+^ and 129 Sv/Ev NHE3^−/−^ housed in a barrier facility did not exhibit macroscopically evident small intestinal pathology (data not shown). Adenomas observed in Apc^Min/+^NHE3^−/−^ mice were limited to the distal 2 cm of the colon. The resulting chimeric background could potentially be responsible for the observed lack of small intestinal polyps. Sødring et al.^45^ back-crossed the Apc^Min/+^ to A/J mice resulting in increased colonic tumor formation, with some of the tumors progressing to carcinomas. Moser et al.^46^ had shown that 129X1B6 Apc^Min/+^ mice had significantly fewer intestinal tumors than did the B6 Apc^Min/+^ mice. This latter observation suggests that 129X1B6 Apc^Min/+^ is more resistant to tumor development and that our F2 cross between BL6 and 129 mice may have actually resulted in underappreciation of the negative effects of NHE3 deficiency, and the observed colonic tumorigenicity would have been further enhanced if the cross were maintained on pure B6 background.

Loss of NHE3, normally expressed in the colonic surface epithelium, has likely pleiotropic effects on tumor growth. We have previously reported that NHE3^−/−^ mice develop dysbiosis reminiscent of human ulcerative colitis, and that susceptibility to mucosal inflammation can be transferred via fecal microbiome transplant into germ-free mice^14,15,20,30^. Development of adenomas in Apc^Min/+^ mice is also influenced by the resident microbiota, although contrary observations have been reported for germ-free and antibiotic-depleted mice^47–49^. In Apc^Min/+^IL10^−/−^ mice, when inflammation compounds Apc haploinsufficiency, bacteria were indispensable for colon carcinogenesis^28^. In our study, the effects of NHE3 deficiency was the dominant factor influencing both fecal and mucosal microbiota. Apc^Min/+^ genotype further influenced microbiota composition in NHE3^−/−^ mice. While increased *Ruminococcaceae*, *Lachnospiraceae, Ruminococcus, Oscillibacter* genera was consistent with reports in CRC patients^50,51^, it was surprising to observe increase relative abundance of *Acetatifactor* and *Lactobacillus*, both suggested to protect from colon cancer development. We found that *Erysipelatoclostridium* was highly positively correlated with increased IL-22, TNFα, NOS2, and IL-6 expression in Apc^Min/+^NHE3^−/−^ mice, and negatively correlated with SCFA producers (e.g. *Ruminiclostridium, Acetatifactor*, *Anaerosporobacter, Roseburia, Eisenbergiella*, and others). These and other reported findings from Spearman correlation analysis supports the notion that the mucosal inflammation developed as a consequence of NHE3 loss contributes to ecological shifts that may favor tumor growth. The comparison of mucosal-adherent bacteria at tumor sites vs. unaffected mucosa in Apc^Min/+^NHE3^−/−^ mice yielded relatively low-confidence data pointing to decreased relative abundances of *Alistipes, Ruminiclostridium_5, Muribaculum, and Erisipelatoclostridium.* Relatively little is known about the role of these genera in the pathogenesis of colonic inflammation and CRC. *Alistipes* have been suggested to act as a pathobiont and *A. finegoldii* promoted right sided CRC via the IL-6/STAT 3 pathway^52^. However, the effects of *Alistipes* spp. may be context-dependent as others reported immunomodulatory and anti-inflammatory effects as well^53^. Based on PICRUSt2 algorithm prediction, the effects of NHE3 deficiency again had the dominant effect and comparison of NHE3^−/−^ vs WT (both on Apc^+/+^ background) yielded similar sets of over- and under-represented metabolic pathways. One notable distinction was a dramatic loss of bacteria capable of glucose and glucose-1-phospate degradation in Apc^Min/+^NHE3^−/−^ mice. This may represent an ecological shift away from utilization of glucose as a sole/main source of carbon and energy. This may increase luminal glucose availability to tumor cells to facilitate aerobic glycolysis (Warburg effect) and tumor growth.

Based on the greatly elevated levels of pSer^139^ γ-H2AX in the tumor surface epithelium of Apc^Min/+^NHE3^−/−^ mice, we can also speculate that loss of NHE3 in colorectal tumors leads to escalated DNA damage and local changes in surface barrier integrity. This may, in turn, allow for the influx of bacterial products into the tumor stroma to elicit stronger chronic inflammatory responses and promote tumor growth. In support of this notion, Wang et al.^34^ showed that excess of IL-22 drives expression of NOS2 (both elevated in Apc^Min/+^NHE3^−/−^ mice) and the resulting NO_x_ species drive DNA damage and promote tumor growth in a model of colitis-associated CRC. Further work would be required to verify the causal involvement of IL-22, a cytokine with pleiotropic effects in the gut mucosa, in colonic tumor formation in the absence of NHE3.

It was plausible that in addition to extrinsic cues precipitated by NHE3 deficiency (microbiota, inflammatory cytokines), loss of NHE3 expression and activity in transformed epithelium could contribute to the observed tumor growth in Apc^Min/+^NHE3^−/−^ mice. SK-CO15 cells were chosen as a well characterized in vitro model^39^, which allowed us to study the consequences of NHE3 loss in colon cancer epithelia. Partial loss of NHE3 expression and activity in human colonic cancer cells let to a decreased resting pHi, and to paradoxically enhanced cancer cell division, sensitivity to DNA damage, and reduced baseline apoptosis and ECM adhesion. This cell behavior is consistent with intrinsic roles of NHE3 in the control of these fundamental aspects of epithelial cancer cell biology, but seemingly contradicts the studies with cells where NHE1 is the predominant Na^+^/H^+^ exchanger that regulates cellular pH. We believe that our findings need to be interpreted not in the broad context of all cancers, but rather in the local context of colonic surface epithelium which, when untransformed, expresses high levels of apical NHE3. In this microenvironment, loss of NHE3 may facilitate DNA damage in a manner similar to that reported for NHE1 in Barrett’s esophagus (BE). NHE1 expression is increased in patients with GERD and BE^54,55^, where it provides a defensive mechanism to manage the acute and chronic acid overload. Bile acids in reflux chyme reduce the ability of the cells to control their pHi by NO-mediated NHE1 inhibition, and lead to increased DNA damage and potentially cancer progression^55^. It is plausible that in the colonic surface epithelium, NHE3 plays similar functions and that its loss due to cancer-related transformation and de-differentiation makes cells more susceptible to genotoxic effects of cytokines, reactive oxygen and nitrogen species. It is plausible that the observed increase in the expression of topoisomerases 2a and in NHE3kd SK-CO15 cells with decreased pHi could contribute to the observed effects. TOP2 is known to be abnormally activated by acidic pH to potently induce DNA damage and cytotoxicity in tumor cells^41^. In conjunction with the effects on colonic microbiota, loss of NHE3 expression and activity likely contributes to local inflammatory response and tumor growth.

## Materials and methods

### Animals and study design

All mice were kept in a specific pathogen free (SPF) barrier animal facility at the Bio5 Institute, University of Arizona. Animal protocol and all procedures were approved by the University of Arizona Animal Care and the Use Committee (#07-126). To generate F1 hybrid mice, males C56BL/6J-Apc^Min^ (The Jackson Laboratory) were cross-bred with females 129S6/SvEv-NHE3^+/−^. Inbreeding the F1 generation C56BL/6Jx129S/SvEv-Apc^Min/+^NHE3^+/−^ males with C56BL/6Jx129S/SvEv-NHE3^+/−^ females generated the F2 generation of hybrids (C56BL/6Jx129S/SvEv) NHE3^+/+^, NHE3^−/−^, Apc^Min/+^NHE3^+/+^, and Apc^Min/+^NHE3^−/−^ mice. Only F2 mice were used for further experiments. All mice were genotyped at age of 3 week, for Apc^Min/+^ allele according to the protocol of the Jackson Laboratory, and for NHE3 allele using our standardized genotyping method^15^. All mice were kept in individually ventilated cages, fed with Teklad Global Diet 2019 with a protein content of 19% and fat 9%, and with ad libitum water access.

Mice were sacrificed by CO_2_ inhalation followed by cervical dislocation. The fecal pellets from distal colon were collected for microbiome analysis and snap-frozen. Then the colon was resected, flushed with cold PBS, and open longitudinally for quantification of polyp/tumor burden. Half of the distal colon was paraformaldehyde fixed for histological evaluation. The other half of the distal colon was snap-frozen for DNA and RNA extraction to evaluate microbiome associated with colon tissue and the expression of selected genes involved in inflammation.

### Histological evaluation and pathology scoring

Tissues for histological evaluation were fixed in 10% buffered formalin (Fisher Scientific). After a 24-h incubation at 4 °C, formalin was replaced with 70% ethanol and samples were submitted to the histology laboratory at the University Animal Care, University of Arizona. For histological assessment the 5 mm thick sections were hematoxylin and eosin (H&E) stained and assessed by an experienced pathologist blinded to the study design based on lesion scoring criteria for mouse intestinal lesions modified from Burich et al.^31^ Colitis score was calculated in non-cancerous regions of the distal colon and is a summary of mucosal proliferation score (scale 0–2), inflammation (scale 0–3), and extent of inflammation or proliferation (scale 0–2) for a maximum score of 7. For correlation of microbiota with objectively measured mucosal inflammation, inflammation score was calculated as a sum of the fold change of analyzed cytokine expression levels (from RT-qPCR results, see below) when compared to wild-type mice. The summarized values were binned as follow: 1 (sum_range 0–7), 2 (sum_range 7–14), 3 (sum_range 15–20), 4 (sum_range 20–50), 5 (sum_range 51–100), 6 (sum > 100) where 1—no inflammation to 6—severe inflammation.

### DNA/RNA extraction

The mechanical disruption of all samples was performed with the high performance, high throughput TissueLyser II (Mo Bio Laboratories) at 4 °C, 2 times 10 min at 20 Hz speed. Total DNA from fecal samples was purified using the high throughput PowerSoil 96 well DNA Isolation Kit (Mo Bio Laboratories) according to a manufacturer protocol. Total DNA and total RNA from distal colon was purified using AllPrep DNA/RNA Mini Kit (Qiagen) with an antifoaming reagent DX (Qiagen) added to a lysis buffer. RNA from SK-CO15 cells was prepared using Trizol (Invitrogen) extraction.

### Microbiome analysis

Microbiome analysis was done based on the V4 hypervariability region of the 16S rRNA gene. Briefly, the V4 fragment was amplified from each sample using 515F and 806R primers. Both primers consist of the regions specific for the amplified fragment, Illumina adapters, and fragments for sequencing primers. Additionally, 806R primer contain a 12 nucleotide Golay barcode as described previously^56^. A unique barcode was use for every sample. The amplicons were checked on the agarose gel for their quality and potential nonspecific PCR products. The concentration of amplicons was determined using Quant-iT PicoGreen ds DNA Assay Kit (Molecular Probes Life Technologies) on 96 well black plates with SpectraMax M3 plate reader (Molecular Devices). Equal amount of DNA from each PCR reaction/sample was pooled and 500 μl of the pooled library was run on 2% agarose gel. The ~ 400 bp band was cut out from the gel and DNA was cleaned using UltraClean DNA Purification Kit (Mo Bio Laboratories, Inc) followed by UltraClean PCR Clean-up Kit (Mo Bio Laboratories, Inc). Cleaned library was quantified with the KAPA Library Quantification Kit (KAPA Biosystems), then the library was diluted to 4 nM and denatured with 0.2 N NaOH by mixing 1 part of the library with 1 part of NaOH. The final concentration of 7 pM was sequenced on MiSeq Illumina platform (M02149, with the MiSeq Control Software v 2.5.0.5). Due to the limited sequence diversity among 16S rRNA amplicons, 5% of the PhiX control library (Illumina) was added to the run. The PhiX control library was denatured and diluted the same way as the experimental library. The pair-ended (2 × 150 bp) sequencing was done using MiSeq Reagent Kit v2 (Illumina).

The data were analyzed as described previously^57^. Briefly, the fastq files were demultiplexed with the *idemp* script (https://github.com/yhwu/idemp), filtering, dereplication, chimera identification, and merging of paired-end reads were performed with *dada2* R package^58^. The ASVs taxonomy was assigned using the Ribosomal Database Project (RDP) classifier^59^ against SILVA database (the latest release, currently release 132^60^, https://www.arb-silva.de/documentation/release-132/). *vegan* R package was used to analyze microbial community (https://CRAN.R-project.org/package=vegan)^61^. DESeq2 package^62^ was used to determine statistically significant different taxa between groups. Results were visualized with *ggplot2* package. Spearman’s rank correlation between genera and pro-inflammatory cytokines levels was calculated with *cor* function form the *stats* package (a part of R) and visualized using a visual exploratory tool *corplot* R package^63^. Statistical analysis and visualization for paired samples were performed with *ggpubr* R Package (https://rpkgs.datanovia.com/ggpubr/)^64^.

### Real-time quantitative PCR

TissueScan Colon Cancer cDNA Array III (Origene, Rockville, MD) was used to quantitate NHE3 gene expression by qPCR. The array plate was comprised of cDNA synthesized from RNA isolated from colonic biopsies from 24 matched pairs (48 samples) covering 24-normal, 5-Stage I, 5-IIA, 2-IIB, 2-IIIA, 3-IIIB, 2-IIIC, 1-III, 4-IV colorectal cancer cases. NHE3 and b-actin mRNA expression was analyzed using specific TaqMan primers/probe sets (Invitrogen) and Roche LightCycler96. In mice, expression of selected genes was analyzed by real-time qPCR. Total RNA (500 ng) purified from previously snap-frozen distal colon tissue (for details see “DNA/RNA Extraction” section) was reverse transcribed with SensiFAST cDNA Synthesis Kit (Bioline) then 1 ml of the reaction was used for real-time qPCR with SensiFast Probe No-ROX Mix (Bioline), commercially available primers from Invitrogen, and Roche LightCycler96. All results were normalized to an endogenous reference GAPDH gene and to a calibrator calculated from Ct values from the control group.

### Immunofluorescent staining

Dissected tissues were fixed overnight in 10% neutral buffered formalin (Fisher Scientific, Tustin, CA, USA) and then stored in 70% ethanol. The tissues were submitted to the University Animal Care Pathology Services, University of Arizona for paraffin embedding and sectioning. Five mm thick sections were deparaffinized by incubation for 5–8 min at 60 °C on the heat block, followed by two 10 min washes in xylene. Subsequently the sections were rehydrated in a series of the gradually reduced ethanol concentration from absolute ethanol (10 min), 90% (8 min), 80% (5 min), 70% (5 min), and then incubation in water (2 × 5 min). Antigen retrieval was conducted in Target Retrieval Solution Citrate pH 6 (DakoCytomation, Denmark) and then slides were washed in water for 5 min. Slides were incubated in blocking buffer (5%BSA or normal goat serum, 0.3% Triton X-100 in PBS) at room temperature for 1 h. Specimens were incubated overnight at 4 °C with primary antibodies (Table 2) in PBS supplemented with 1% normal goat serum and 0.3% Triton X-100 at the following dilutions: 1:200 for anti-γ-H2AX, anti-E-cadherin, ati-pStat3^Tyr705^, and1:400 for NOS2. The next day, slides were washed three times (5 min each) with the same buffer and incubated with appropriate labeled secondary antibodies (in all cases at 1:400 dilution) for 1 h at room temperature. Slides were washed in antibody buffer followed by 3 washes in PBS. DAPI (1 μg/ml final concentration; MP Biomedicals) was added to the last wash as a nuclear counterstain and slides were mounted in antifade mounting medium. Imaging was done using Olympus FLUOVIEW FVi10 confocal microscope. Identical image capture settings were used for all four genotypes.

**Table 2:** Antibodies used in this work. IF-immunofluorescence, *WB* Western Blot.

Antibody	Vendor	Catalog #	Application
γ-H2AX (pSer139)	Cell signaling	9718	IHF
E-cadherin (CD324)	BD pharmingen	567053	IHF
iNOS	Cell signaling	13120	IHF
Phospho-Stat3 (Tyr705)	Cell signaling	9145	IHF
Anti-mouse IgG, HRP linked	Cell signaling	7076	WB
GAPDH	Thermo-Fisher Scientific	MA1-16757	WB
NHE3 (SLC9A3)	Custom (rabbit serum)	Ref.^67^	WB
AlexaFluor 647	Thermo-Fisher Scientific	A-21245	IHF

### Characterization of SK-CO15 cells and NHE3 knockdown (NHE3kd)

SK-CO15 human adenocarcinoma cells were provided by Dr. Asma Nusrat (University of Michigan). SK-CO15 cells, by various morphological and biochemical criteria, displays a pattern of differentiation close to colonic intestinal epithelia with proper polarization and proper protein sorting to the apical and basolateral domains^65^. Mycoplasma-free SK-CO15 cells were cultured in DMEM supplemented with 10% FBS, penicillin (50 mU/ml), streptomycin (50 μg/ml), 1 mM sodium pyruvate, 15 mM HEPES, and 1 × nonessential amino acids. Genomic DNA from SK-CO15 and T84 cells (the latter as control cells which do not express NHE3), along with a synthetic Quantitative Multiplex Reference Standard (gDNA; Horizon Discovery, Waterbeach, United Kingdom; cat. #HD701) was used for targeted sequencing using TruSight Tumor 15 assay (Illumina) covering mutations in 15 solid tumor-related genes (AKT1, GNA11, NRAS, BRAF, GNAQ, PDGFRA, EGFR, KIT, PIK3CA, ERBB2, KRAS, RET, FOXL2, MET, and TP53).

Cells were transduced with lentiviral particles with feline immunodeficiency (FIV)-based plasmid psiLv-U6 (GeneCopoeia; Rockville, MD) with a clone set of four shRNA sequences specifically targeting human *SLC9A3* transcript (GGACAGATCGGGCACAATTAT; GCTACAGCAGTACCTGTACAA; GGCTCAACCAGAACAAGAAGG; GCTAGTGTCACCAAGGACACA) placed under the control of U6 promoter. None of them had any detectable homology with other human Na^+^/H^+^ exchangers. Lentiviral particles with scrambled shRNA were used as control. psiLv-U6 carries EGFP under the control of CMV promoter and puromycin resistance gene under the control of phosphoglycerate kinase (hPGK) promoter. After transduction, cells were flow-sorted based on high EGFP expression and selected in media with puromycin (0.5 μg/ml). Cells were maintained as mixed population without clonal selection to avoid bias.

### Microarray profiling of gene expression in SK-CO15 NHE3kd cells

Amplified and biotinylated sense-stranded DNA targets were generated from total RNA isolated from control and NHE3kd SK-CO15 cells (n = 3 in each group) using GeneChip^®^ WT PLUS Reagent kit (Affymetrix) and hybridized to GeneChip™ Human Gene 2.0 ST Arrays (Affymetrix). Gene expression analysis was performed using GeneSpring GX software (Agilent Technologies, Santa Clara, CA, USA). Data were processed using the RMA16 summarization algorithm and normalized against the mean of control samples for presentation purposes. Statistical analysis was performed using built-in tools, including normalized t test with Benjamini–Hochberg multiple testing correction. Gene ontology (GO) functional annotation analysis was performed either with GeneSpring or using the Database for Annotation, Visualization, and Integrated Discovery (DAVID) online tool^66^.

### Western blotting

Control or NHE3kd SK-CO15 cells were lysed and homogenized in radioimmunoprecipitation assay buffer (RIPA) containing 50 mM Tris–HCl (pH 8.0), 150 mM NaCl, 1% NP-40, 0.1% SDS, 0.5% Na-deoxycholate and Halt protease and phosphatase inhibitor cocktail (ThermoFisher Scientific). Protein concentration was determined using a BCA Protein Assay (ThermoFisher Scientific) and samples were denatured and reduced in SDS-PAGE sample buffer (supplemented with 10% β-mercaptoethanol) by heating to 95 °C for 5 min. Samples (20 μg/well) were separated on a 10% Tris–Glycine protein gel (BioRad) for higher molecular weight protein detection (> 30 kDa) and transferred onto nitrocellulose membranes. The blots were probed with primary monoclonal rabbit anti-P53 antibody (clone 7F5, Cell Signaling Technologies) diluted 1:1000 in 5% nonfat milk or BSA) or custom anti-NHE3 antiserum^67^ overnight at 4 °C, then with an HRP-conjugated goat anti-rabbit HRP-conjugated secondary antibody (1:2500) (Cell Signaling). Blots were developed using ECL Western Blotting Substrate (Thermo Scientific) and chemiluminescence was detected using the Syngene G:BOX Imaging System and GeneSys software (Syngene, Frederick, MD).

### γ-H2AX ELISA

Lysates of control or NHE3kd SK-CO15 cells were assayed for human phospho-histone H2AX by ELISA using a commercially available kit (R&D Systems, Minneapolis, MN; cat. # DYC2288) according to manufacturer’s instructions.

### Alkaline comet assay

To assess lack of NHE3 on the extent of the DNA damage in SK-CO15 cells the alkaline comet assay was conducted as described previously^68,69^. Control and NHE3kd cells were grown on 6 well plates until 80–90% confluent followed by treatment with 10 mM camptothecin in DMSO, 20 mM freshly prepared hydrogen peroxide, or DMSO (vehicle control). After 1 h, the cells were detached with 0.005% trypsin, washed in Ca^2+^/Mg^2+^-free PBS, and 10,000 cells were resuspended in 500 ml 1% Low Melting Point Agarose (Promega). The samples were loaded onto 1% Normal Melting Agarose-coated microscope slides and allowed to gel. Slides were treated with the Lysis Buffer (2.5 M NaCl, 100 mM EDTA, 10 mM Trizma Base, 0.1% Triton X-100, pH 10) for 2 h at 4 °C followed by 3 washes, 20 min each with alkaline electrophoresis buffer (300 mM NaOH, 1 mM EDTA, pH > 13) allowing DNA unwinding and denaturation. Slides were placed on the horizontal gel box filled with fresh electrophoresis buffer and electrophorese the slides for 20 min at 25 V, 300 mA. The slides were removed and neutralizing buffer (0.4 M Tris, pH 7.5) was applied dropwise followed by staining for 5 min with 2 mg/ml DAPI and wash with distilled water to remove excess stain. The slides were visualized using EVOS^®^ FL Auto Imaging System (ThermoFisher Scientific).

### Cell proliferation

Cell proliferation was measured using CellTiter 96 AQueous One Solution Cell Proliferation Assay (MTS) (Promega; Madison, WI) according to the manufacturer’s protocol.

### Apoptosis

Baseline rate of apoptosis in control and NHE3kd cells was assessed using Caspase-Glo 3/7 Assay System (Promega) according to the manufacturer’s protocol.

### ECM adhesion assay

ECM adhesion assay of control or NHE3kd cells (2 × 10^5^ cells/well, 2-h adhesion time) was performed using colorimetric ECM Cell Adhesion Array kit (Millipore, Cat. #ECM540) according to the manufacturer’s protocol.

### Ethics approval

All experiments were performed in accordance with relevant guidelines and regulations. Animal experiments were approved by the institutional committee for animal care and use of the University of Arizona (protocol 07-126; Kiela). Patient consent for publication**:** Not required. The study was carried out in compliance with the ARRIVE guidelines.

## Supplementary Information

Supplementary Information.

## Data Availability

All raw data from microbiome analysis has been submitted to the NCBI Sequence Read Archive (SRA) database (BioProject ID PRJNA852254; http://www.ncbi.nlm.nih.gov/bioproject/852254). Transcriptome profiling data has been submitted to NCBI GEO database (accession # GSE184326; https://www.ncbi.nlm.nih.gov/geo/query/acc.cgi?acc=GSE184326).
