# Supplementary material for: Decreased NHE3 expression in colon cancer is associated with DNA damage, increased inflammation and tumor growth
Source: Sci Rep. 2022 Aug 30;12:14725. doi: 10.1038/s41598-022-19091-x (PMC9427942; doi:10.1038/s41598-022-19091-x)
Supplement: Supplementary file 1 — Supplementary Information. [file 41598_2022_19091_MOESM1_ESM.pdf]

DECREASED NHE3 EXPRESSION IN COLON CANCER IS ASSOCIATED WITH DNA DAMAGE, INCREASED INFLAMMATION AND TUMOR GROWTH

Daniel Laubitz[1*], Michael A. Gurney[1*], Monica Midura-Kiela[1], Christy Clutter[1], David G Besselsen[2], Hao Chen[3], Fayez K. Ghishan[1#], and Pawel R. Kiela[1,4#].

[1]Department of Pediatrics, Steele Children's Research Center. University of Arizona College of Medicine, Tucson, AZ, [2]University Animal Care, University of Arizona, Tucson, Arizona
[3]Department of Pathology, University of Texas Southwestern Medical Center, Dallas, TX.
[4] Department of Immunobiology, University of Arizona College of Medicine, Tucson, AZ

SUPPLEMETAL INFORMATION

(Figures S1-S6 and Supplemental Table S1)

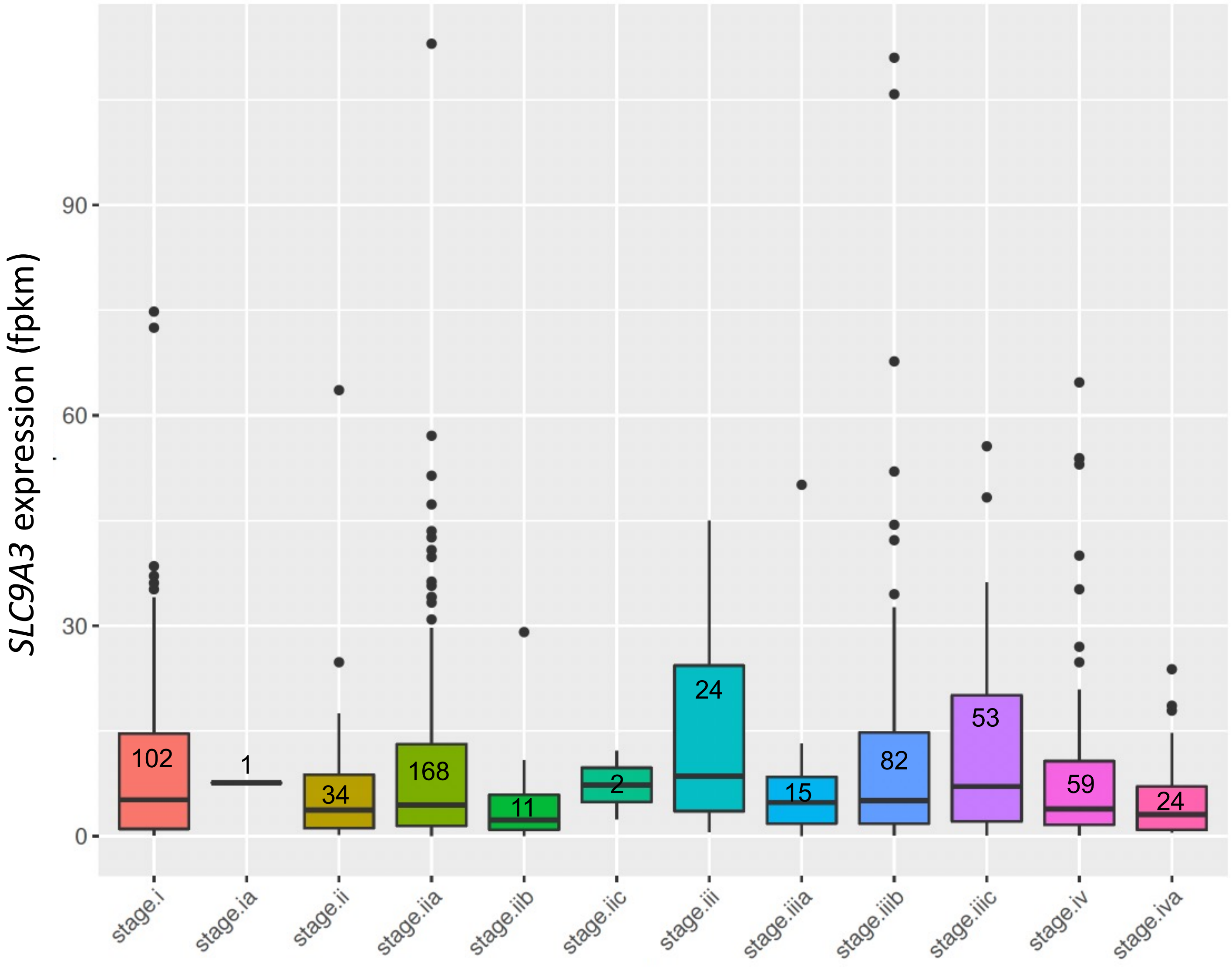

Figure S1: Expression of NHE3 is not dependent on the stage of colorectal cancer. The Y-axis shows the expression levels (FPKM; Fragments Per Kilobase of transcript per Million mapped reads) of the SLC9A3/NHE3 gene in the tumor tissue at the time of diagnosis. Y-axis depicts tumor stage. Number of patients in each stage bin indicated in each bar. No statistical differences were observed. The results shown here are in whole based upon data generated by the TCGA Research Network: https://www.cancer.gov/tcga.

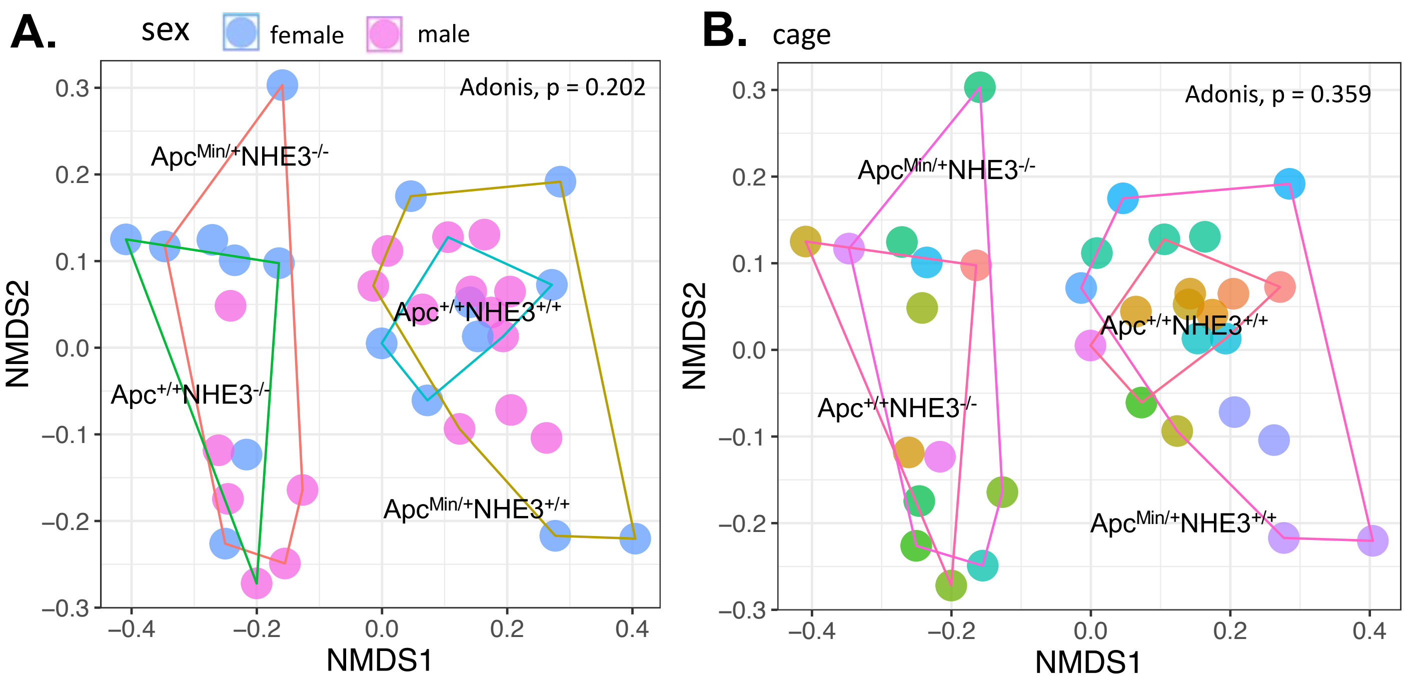

Figure S2: Non-metric Multidimensional Scaling (NMDS) analysis based on Bray-Curtis distances analyzed on context of sex (**A**) and housing/cage (**B**). Both factors did not have a significant impact on changes in gut microbiota as determined with the Adonis test. Each dot represent samples from individual mice. The color of the dots represent sex (**A**) or the individual mouse cages (**B**). Polygons connect points within the same cluster representing individual genotypes as indicated.

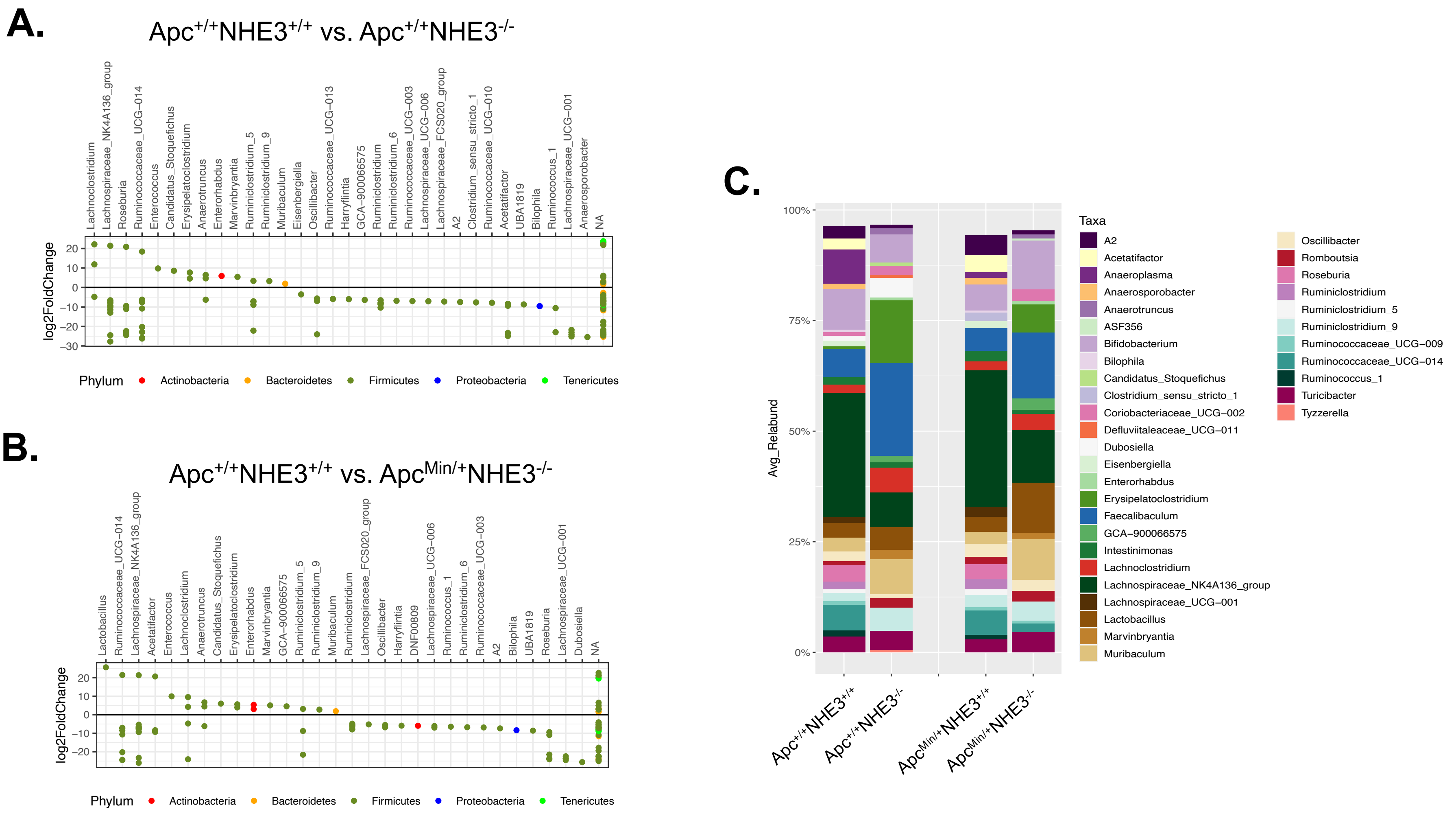

Figure S3: Differential abundance analysis at the genus level using DESeq2 tool between wild type ($Apc^{+/+}NHE3^{+/+}$) mice vs. $Apc^{+/+}NHE3^{-/-}$ mice (**A**) or $Apc^{Min/+}NHE3^{-/-}$ mice (**B**). Each dot represents an individual ASV. The color of the dots represents a specific phylum (red – Actinobacteria, yellow – Bacteroidetes, dark green – Firmicutes, blue – Proteobacteria, and bright green Tenericutes). For the presentation, only significantly different taxa (Wald significance test <0.05) with at least 2-fold change (absolute value of log2FoldChange > 1) were selected. (**C**) Microbial composition (relative abundance) at the genus level in all four analyzed genotypes. For the presentation, genera with relative abundance lower than 0.5% were removed. Figure for panel C was generated ggplot2 package for R (ver 3.3.6; https://ggplot2.tidyverse.org).

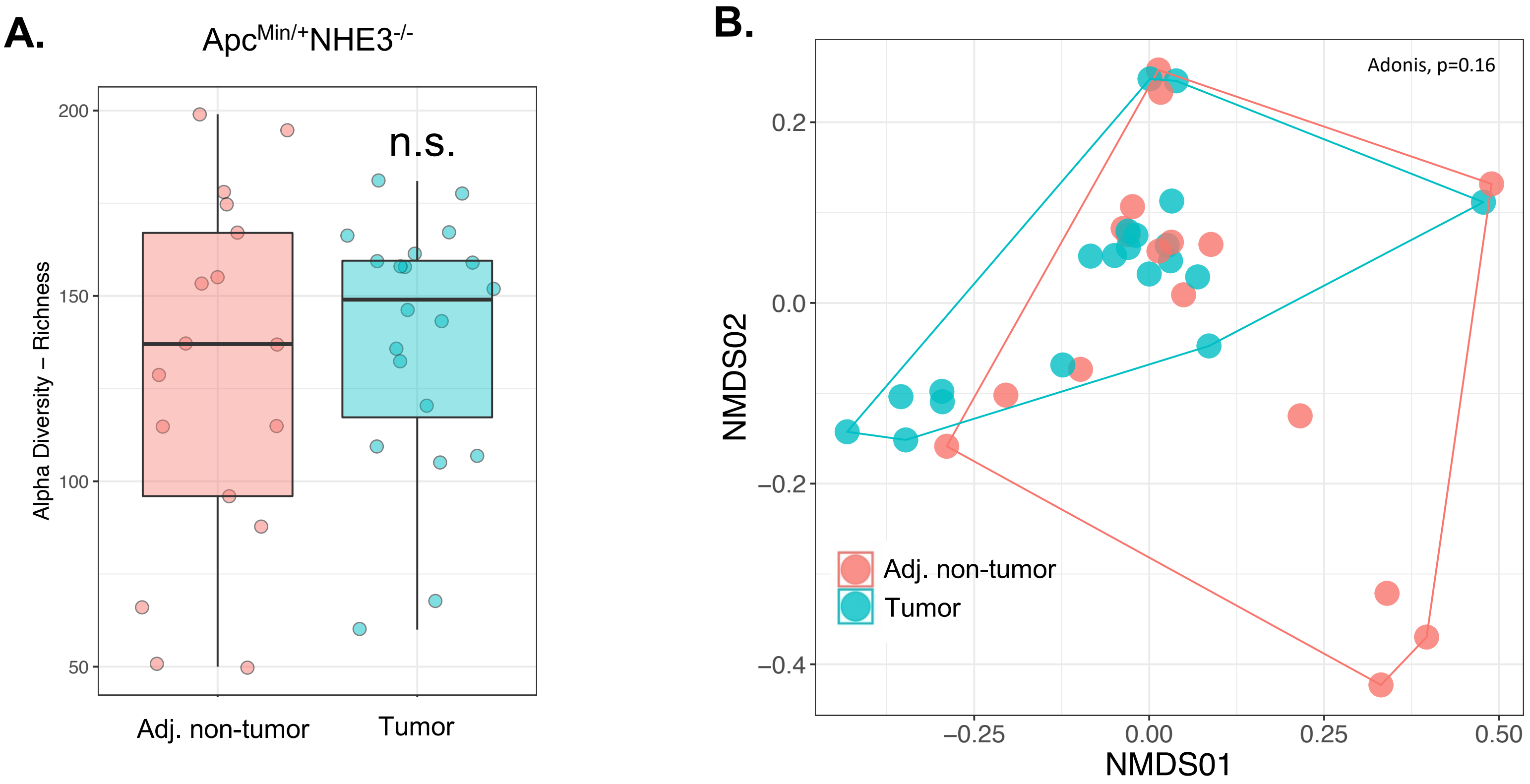

Figure S4AB: **Mucosal adherent microbiota at the colonic tumor site and adjacent non-tumor (Adj. non-tumor) site are not significantly different** as analyzed by richness, α-diversity index (**A**) and Non-metric Multidimensional Scaling (NMDS) analysis based on Bray-Curtis distances, a β-diversity measure (**B**).

C.

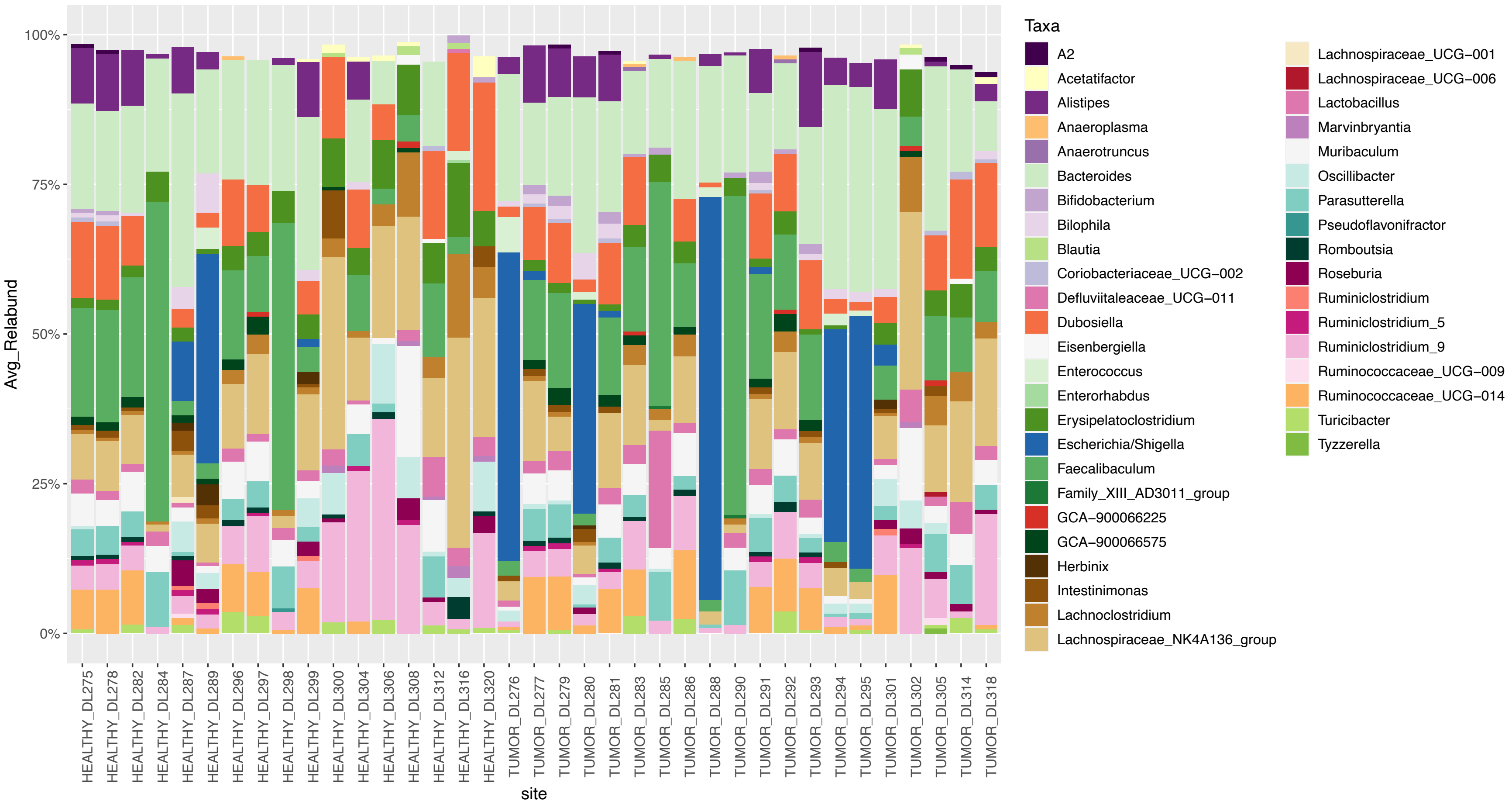

Figure S4C: **Mucosal adherent microbiota at the colonic tumor site and adjacent non-tumor site.** (**C**) Microbial composition (relative abundance) of individual samples at the genus level in samples collected from tumors and adjacent non-tumor tissues (Adj. non-tumor, designated as HEALTHY). For the presentation genera with relative abundance lower than 0.5% were removed. The samples are labeled as the collection site followed by a sample ID. Figure for panel C was generated ggplot2 package for R (ver 3.3.6; https://ggplot2.tidyverse.org).

D.

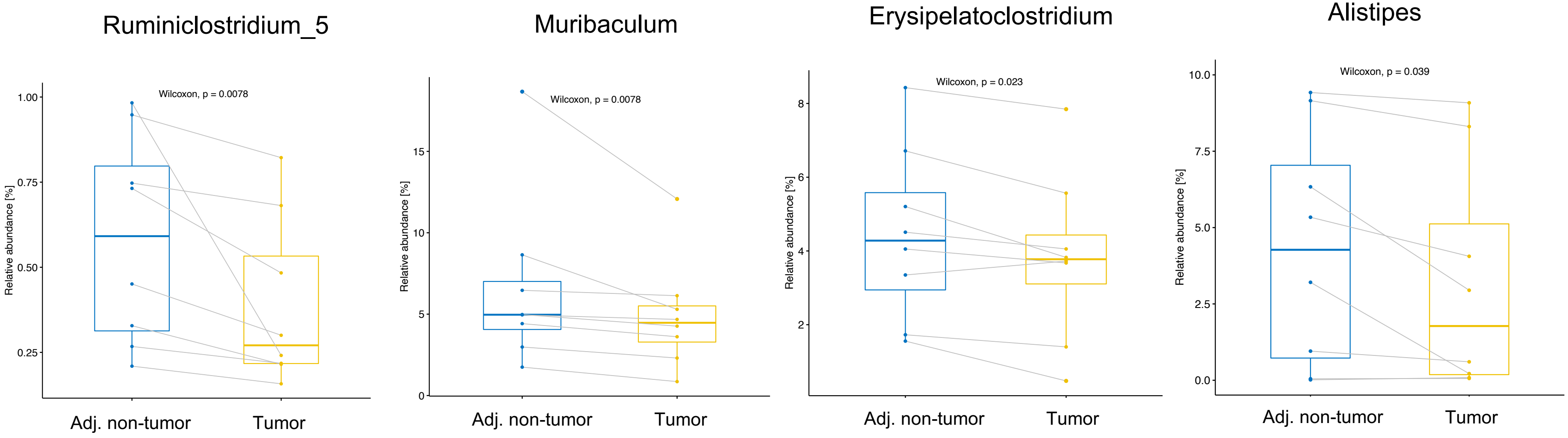

taxa	p	p.adj	p.signif	method
Alistipes	0.0390625	0.16	*	Wilcoxon
Erysipelatoclostridium	0.0234375	0.094	*	Wilcoxon
Muribaculum	0.0078125	0.031	**	Wilcoxon
Ruminiclostridium_5	0.0078125	0.031	**	Wilcoxon

Figure S4D: **Mucosal adherent microbiota at the colonic tumor site and adjacent non-tumor site.** Paired analysis of microbial composition between tumor and adjacent non-tumor (Adj. non-tumor) indicated four significantly different taxa. Paired Wilcoxon signed-rank test was used.

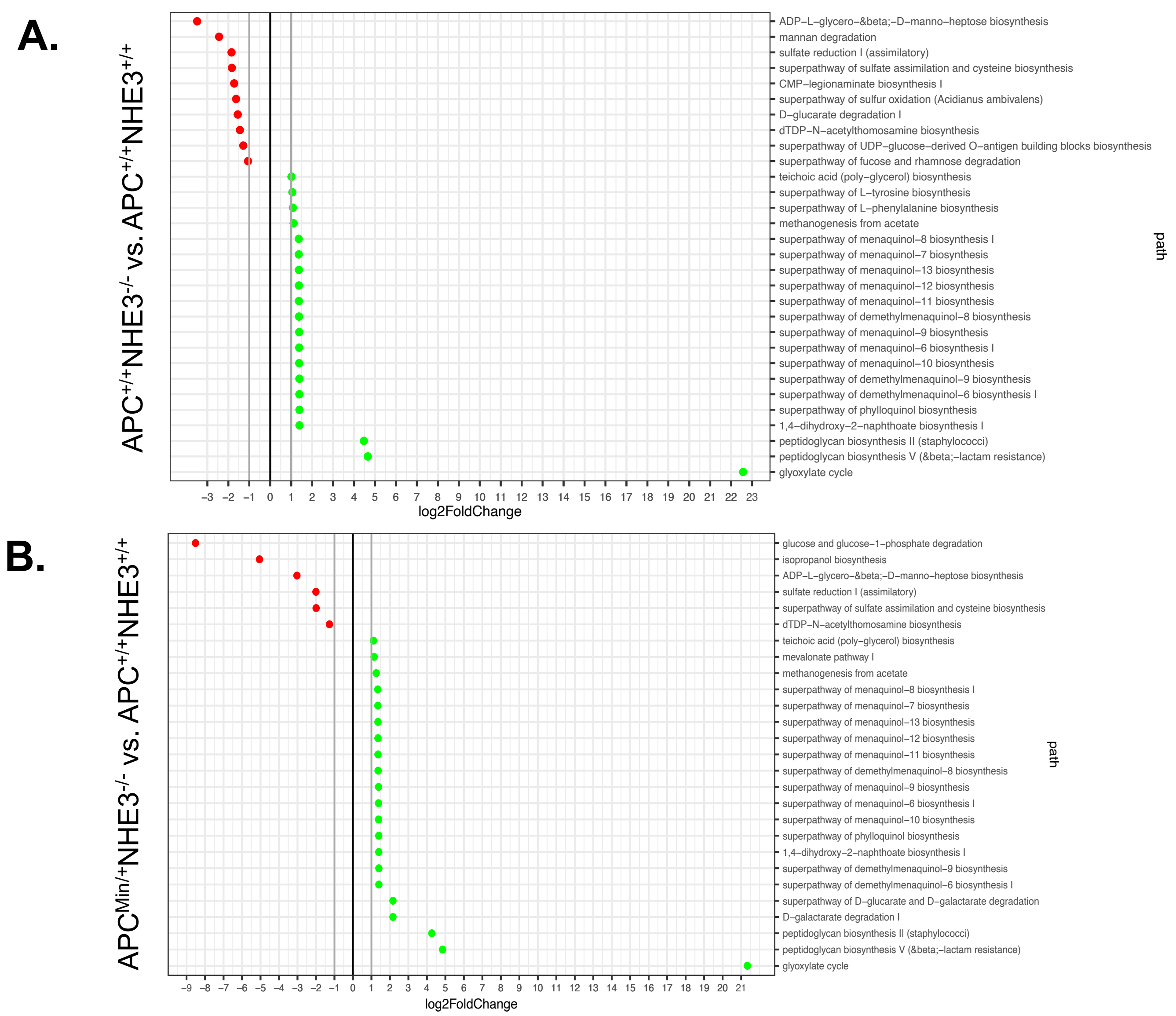

Figure S5: PICRUSt2 functional metabolic prediction of gut microbial population between (**A**) APC$^{+/+}$NHE3$^{+/+}$ vs. APC$^{+/+}$NHE3$^{-/-}$ and (**B**) APC$^{Min/+}$NHE3$^{-/-}$ vs. APC$^{+/+}$NHE3$^{+/+}$. To identify statistically different abundance of the EC pathways and visualized results, DESeq2 pipeline was used. For the presentation, only significantly different taxa (Wald significance test <0.05) with at least 2-fold change (absolute value of log2FoldChange > 1) were selected. Red dots represent decreased abundance and green dots increased abundance of EC metabolic pathways.

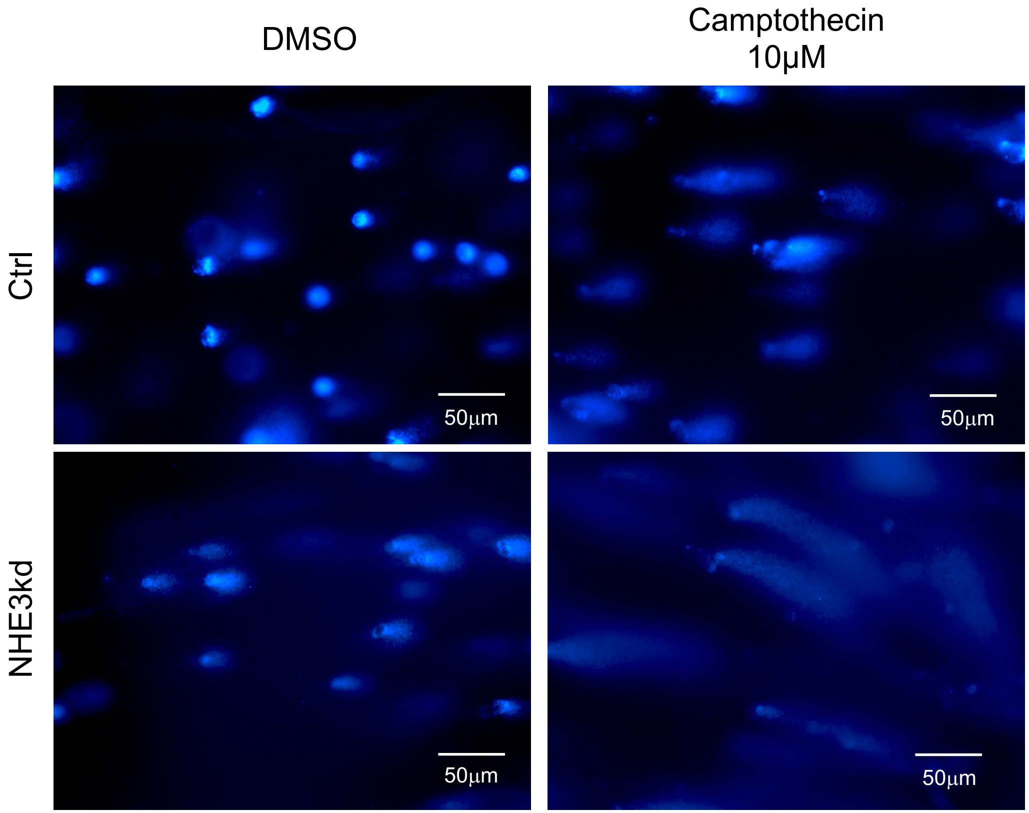

Figure S6: NHE3 knockdown in in SK-CO15 colonic adenocarcinoma cells leads to enhanced susceptibility to DNA damage. Low magnification image of comet assay of Ctrl and NHE3kd SK-CO15 cells treated with and without 10μM camptothecin.

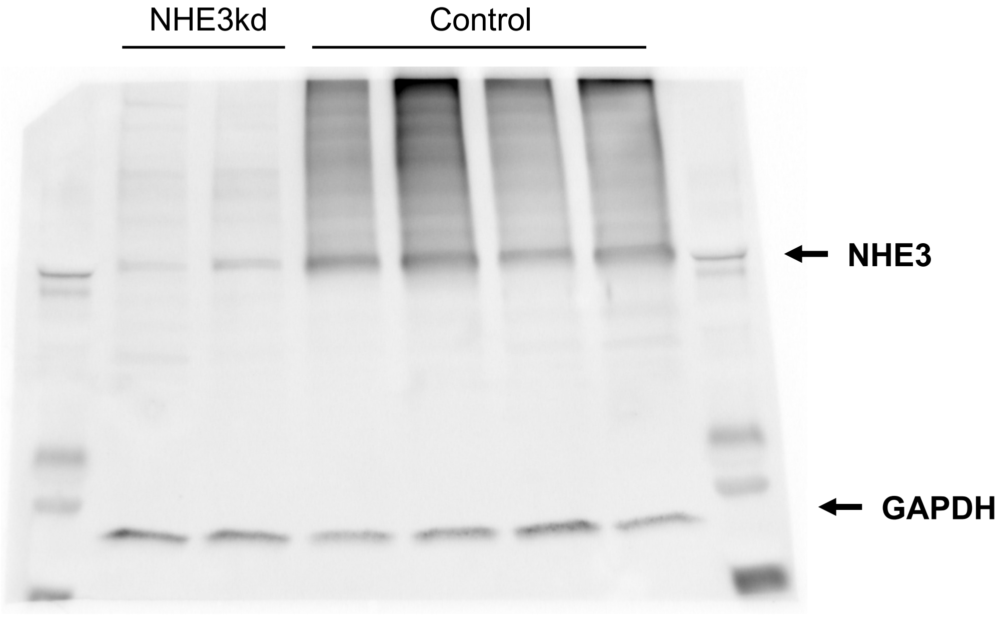

Figure S7: Full capture of Western blot presented in Fig. 5A. Dual detection of NHE3 and GAPDH. Analysis was performed on four different passages of control SK-CO15 cells (scrambled shRNA) and two passages of NHE3kd (NHE3 knock-down).
